# Copper-mediated arylation with arylboronic acids: Facile and modular synthesis of triarylmethanes

**DOI:** 10.3762/bjoc.12.49

**Published:** 2016-03-11

**Authors:** H Surya Prakash Rao, A Veera Bhadra Rao

**Affiliations:** 1 Department of Chemistry, Pondicherry University, Pondicherry 605 014, India Telephone: +914132654411; Fax: +914132656230

**Keywords:** copper, modular synthesis, triarylmethanes

## Abstract

A facile and modular synthesis of triarylmethanes was achieved in good yield via a two-step sequence in which the final step is the copper(II)-catalyzed arylation of diarylmethanols with arylboronic acids. By using this protocol a variety of symmetrical and unsymmetrical triarylmethanes were synthesized. As an application of the newly developed methodology, we demonstrate a high-yielding synthesis of the triarylmethane intermediate towards an anti-breast-cancer drug candidate.

## Introduction

**Figure 1:**
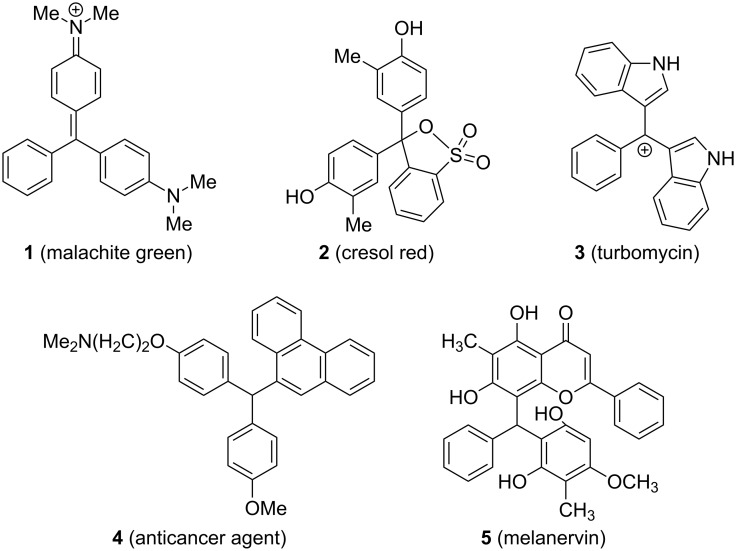
Representative examples of triarylmethanes.

The triarylmethanes form an exclusive group of organic molecules wherein three aryl groups are attached to the central sp^3^-hybridized carbon atom bearing a hydrogen atom [1–4]. Although the group can be restricted to such molecules, many closely related derivatives that have a triarylmethane motif (like those having a heteroatom attached to the central carbon atom or the central carbon is sp^2^ hybridized) have been included in this class [5]. Molecules with a triarylmethane motif are ubiquitous and found mainly in technologically and medicinally relevant molecules like dyes [6–9], pH indicators [10–12], fluorescent probes [13–18] and antibacterial drugs [19]. For example, malachite green (**1**) is a dye, cresol red (**2**) is a pH indicator and turbomycin (**3**) is an antibacterial medicinal drug (Figure 1). Genuine triarylmethane **4**, having three different aryl groups on the central CH, is a proven anti-breast-cancer agent [20]. In addition to **4**, several other triarylmethanes exhibit interesting biological activity, including oestrogen receptor binding affinity [21], inhibition of hepatic cholesterol [22], inhibition of aldose reductase [23], antiproliferative [24], antiviral, cytotoxic [25], antifungal [26], anti-HIV [27–29] and antibacterial activity [30]. Although rare, there are a few natural products, for example, melanervin (**5**), a flavanoid bearing the triarylmethane motif [31–32].

**Scheme 1:**
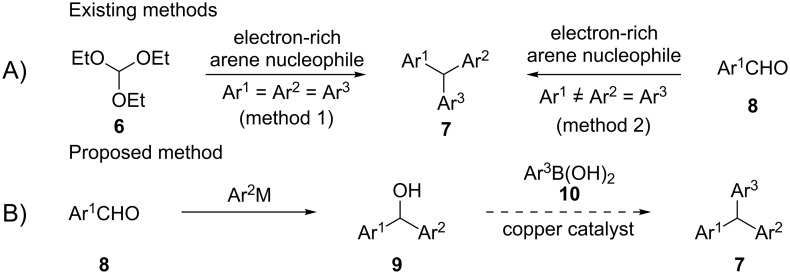
General methods and proposed method for the synthesis of triarylmethanes.

Triarylmethanes are typically synthesized by a Friedel–Crafts-type substitution of the three alkoxy groups in a trialkyl orthoformate (Scheme 1A, method 1) [33–35] or by sequential two-step addition of electron-rich aromatic nucleophiles to activated arene aldehydes followed by substitution of the resulting hydroxy group with another electron-rich aromatic compound (Scheme 1A, method 2) [36–39]. Both of the approaches are limited in scope and suffer from drawbacks such as (a) electron-rich aromatic systems that are required as nucleophiles and therefore, not amenable for the synthesis of triarylmethanes with electron-withdrawing groups, (b) the regioselectivity in the substitution at the aromatic ring that depends on the *ortho*- or *para*-directing nature of the substituent and also by the steric hindrance offered by the substitution, (c) the methods are rarely modular and not suitable for the preparation of triarylmethanes with three different aryl groups, and finaly, (d) Lewis [40–41] or protic acids [42] are required to catalyze the reactions. To overcome the above-mentioned difficulties, many efforts have recently been directed towards transition metal-catalyzed cross-couplings [43–48] or CH arylation followed by an arylative desulfonation [49–50]. The coupling reactions provide an opportunity to install an unactivated aryl group on a carbon bearing two more aryl groups to synthesize the triarylmethane motif. Recently, we reported a copper-catalyzed C–C bond formation by substitution of the labile C(4)SMe group in 4*H*-chromenes or C(3)–OH in isoindolinones with aryl/alkenyl groups by employing the corresponding boronic acids [51–52]. Continuing these efforts, we designed a copper-catalyzed synthesis of a variety of triarylmethanes through substitution of C(sp^3^)–OH in diarylmethanols with arylboronic acids (Scheme 1B). We reasoned that since diarylmethanols with two different aromatic rings can be made by a wide variety of methods [53–54] (e.g., addition of an aryl carbanion to an aryl aldehyde and a further step with a variety of aryl boronic acids), it should be possible to provide a unique opportunity for the modular synthesis of unsymmetrical triarylmethanes. If successful, the method could provide an opportunity for the synthesis of a combinatorial library of the coveted molecules. Herein, we report a copper(II) triflate-catalyzed modular synthesis of triarylmethanes by employing diarylmethanols **9** and arylboronic acids **10**. It is advantageous to employ a base metal catalyst such as copper(II) triflate instead of palladium [55–56] or nickel (Ni) [57] catalysts and to avoid the use of phosphine ligands as it is less expensive and more readily facilitates purification.

## Results and Discussion

We selected the copper-mediated cross-coupling reaction of diphenylmethanol (**9a**) with phenylboronic acid (**10a**) for the synthesis of triphenylmethane (**11a**) to optimize the reaction conditions and catalyst loading. Based on our accrued experience [52], in a first attempt, we employed Cu(OTf)_2_ (10 mol %) in refluxing 1,2-dichlorethane (DCE) to effect C–C coupling, but the reaction provided (phenoxymethylene)dibenzene (**12**) as the only product formed through the C–O coupling (Chan–Lam–Evans coupling product) [58–60] in 64% yield.

**Scheme 2:**
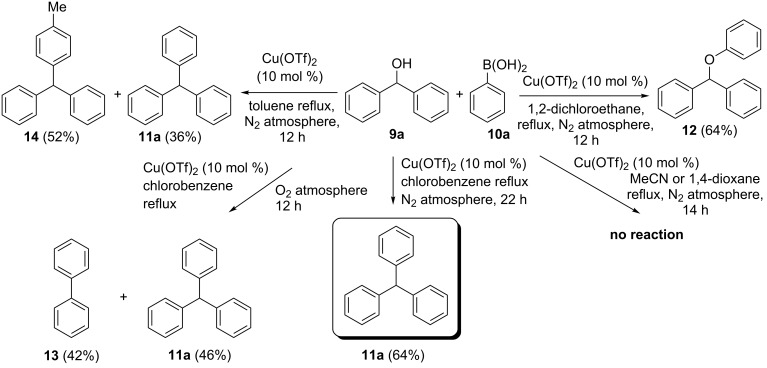
Role of solvent and reaction conditions in the Cu(OTf)_2_-mediated coupling of diphenylmethanol (**9a**) with phenylboronic acid (**10a**) for the preparation of triphenylmethane (**11a**).

To obtain the desired triphenylmethane (**11a**) as the sole product, we screened various alternative solvents (Scheme 2). Of the solvents investigated, toluene gave a mixture of triphenylmethane (**11a**) and the toluene-incorporated product (*p*-tolylmethylene)dibenzene (**14**) in 36% and 52% yield, respectively (Scheme 2). Solvents like acetonitrile (polar, aprotic) and dioxane (oxygenated, aprotic) did not provide the triphenylmethane (**11a**). On the other hand, the higher-boiling, nonpolar chlorobenzene (Scheme 2) at 80 °C provided the coupled product triphenylmethane (**11a**) in 64% yield. When the reaction was conducted under oxygen atmosphere, the yield of **11a** fell to 46%. Under these aerobic conditions, we isolated biphenyl (**13**) generated through homocoupling of phenylboronic acid. Attempts to improve the yield by the use of bases such as Na_2_CO_3_ (10 mol %) and K_2_CO_3_ (10 mol %) were not successful. The yield of triphenylmethane (**11a**) was further reduced in these cases. The base was employed to trap the boric acid, which is likely to be the side product of the reaction. From the above experiments, we concluded that chlorobenzene was the most suitable solvent for the synthesis of triphenylmethane (**11a**).

Next, we turned our attention to evaluate different copper salts to optimize the yield of triphenylmethane (**11a**); these efforts have been summarized in Table 1. We screened various Cu(II) catalysts such as Cu(OAc)_2_ (64%, Table 1, entry 1), Cu(CF_3_COO)_2_ (46%, Table 1, entry 2) Cu(acac)_2_ (36%, Table 1, entry 3), CuSO_4_·5H_2_O (36%, Table 1, entry 4), CuBr_2_ (14%, Table 1, entry 5), CuCl_2_·2H_2_O (24%, Table 1, entry 6) and CuO (no reaction, Table 1, entry 8), which did not provide the desired triphenylmethane (**11a**) in better yield. However, 20 mol % of Cu(OTf)_2_ (Table 1, entry 17) delivered the desired triphenylmethane (**11a**) in good yield (78%) after chromatographic purification. We screened other borderline Lewis acids such as Sc(OTf)_3_, Yb(OTf)_3_, Zn(OTf)_2_ and Fe(OTf)_3_, but the reaction did not afford triphenylmethane (**11a**) at all, which indicated that Cu(II) and not TfOH is responsible for the transformation. Thus, the optimal conditions for the copper-mediated coupling involve heating equimolar amounts of diphenylmethanol (**9a**) and phenylboronic acid (**10a**) in chlorobenzene at 80 °C in the presence of 20 mol % of Cu(OTf)_2_ under a blanket of oxygen-free nitrogen.

**Table 1:** Screening of metal catalysts for the arylation reaction.

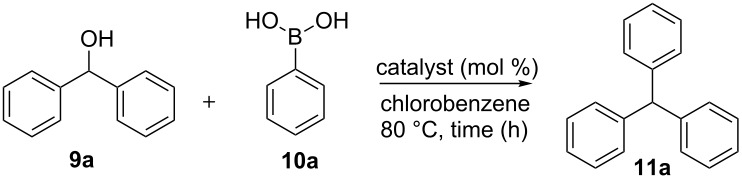				

Entry	Catalyst	Time (h)	Catalyst (mol %)	Yield (%)^a^

1	Cu(OAc)_2_·H_2_O	21	10	64
2	Cu(OOCCF_3_)_2_	18	10	46
3	Cu(acac)_2_	21	10	36
4	CuSO_4_·5H_2_O	19	10	36
5	CuBr_2_	16	10	14
6	CuCl_2_·H_2_O	16	10	24
7	Cu(OTf)_2_	21	10	68
8	CuO	12	10	n.r.
9	CuI	21	10	42
10	CuBr	21	10	14
11	CuCl	19	10	28
12	Cu_2_O	12	10	n.r.
13	Cu(I)BrSMe_2_	12	10	n.r.
14	Cu(PPh_3_)_2_Br	12	10	n.r.
15	CuMeSal	21	10	42
16	CuTc	21	10	27
**17**	**Cu(OTf)_2_**	**18**	**20**	**78**
18	Sc(OTf)_3_	12	10	n.r.
19	Fe(OTf)_3_	12	10	n.r.
20	Zn(OTf)_2_	12	10	n.r.
21	Yb(OTf)_3_	12	10	n.r.

^a^n.r. = no reaction.

**Scheme 3:**
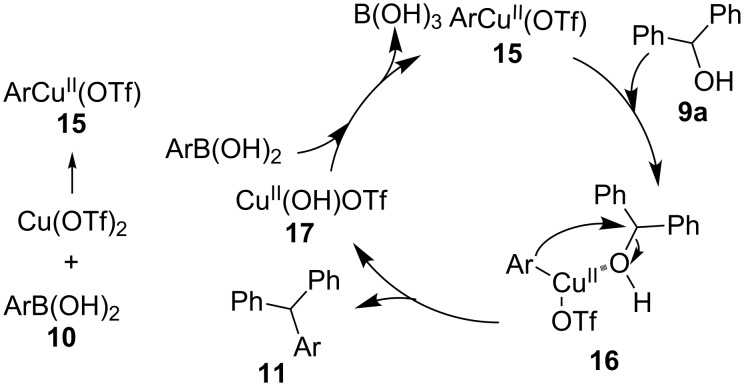
A plausible mechanism for the formation of triarylmethanes **11**.

Based on the above observations, we propose a mechanism for the copper-mediated coupling of phenylboronic acid with diphenylmethanol, leading to triphenylmethane and boric acid (Scheme 3). At the start of the cascade, the first step is the transmetallation of the copper(II) into phenylboronic acid to form reactive PhCu(OTf) (**15**) and B(OH)_2_(OTf) [61]. The intermediate **15** then reacts with diphenylmethanol **9** to provide the intermediate **16**. Formation of the intermediate **16** can be attributed to Lewis acidic characteristics of **15** and Lewis basic characteristics of diphenylmethanol (**9a**). The crucial C–C bond formation with simultaneous C–O bond cleavage subsequently occurs in **16** to give the triarylmethane **11** and copper(OH)(OTf) (**17**). The reaction of **17** with arylboronic acid **10** regenerates **15** and results in the formation of stable boric acid. The driving force for the triarylmethane formation is the generation of a stable C–C bond in **11**, a Cu–O bond in **17**, and boric acid at the cost of weak Ar–Cu and C–OH bonds in **16** and **9**, respectively [52].

With the optimized reaction conditions in hand, we examined the scope of the cross-coupling reaction for the synthesis of a variety of triarylmethanes from diphenylmethanol (**9a**). Ten more arylboronic acids were employed in the coupling reaction and good yields (77–92%) of the corresponding triarylmethanes **11b–k** were realized (Table 2). The arylboronic acids **10b–k** were selected considering their structural diversity and electron density in the aryl ring. Efficient cross-coupling could be noted irrespective of the presence of strongly electron-withdrawing (**10b**,**c** to **11b**,**c**; Table 2, entries 1 and 2), mildly electron-withdrawing (**10d**,**e** to **11d**,**e**; Table 2, entries 3 and 4), strongly electron-donating (**10f** to **11f**; Table 2, entry 5) or mildly electron-donating (**10g** to **11g**; Table 2, entry 6) groups at the C(4) position of the phenyl ring. The robust nature of the protocol was demonstrated by reacting *ortho*-methoxyphenylboronic acid (**10h**) to efficiently generate the desired triarylmethane **11h** (Table 2, entry 7). The transformation showed that apart from the insensitivity towards electronic effects, the copper-mediated cross-coupling reaction is not very sensitive to steric crowding in the neighborhood of the reaction center. Next, we employed heteroaromatic boronic acids, such as furan-2-ylboronic acid (**10i**; Table 2, entry 8), thiophen-2-ylboronic acid (**10j**; Table 2, entry 9) and benzo[*b*]thiophen-2-ylboronic acid (**10k**; Table 2, entry 10) in the coupling reaction and the reactions furnished the corresponding triarylmethanes **11a–k** in excellent yield. The transformations showed that heteroaromatic groups, including those bearing a sulfur atom, react efficiently to provide triarylmethanes.

**Table 2:** Scope of the Cu-catalyzed arylation with various arylboronic acids.

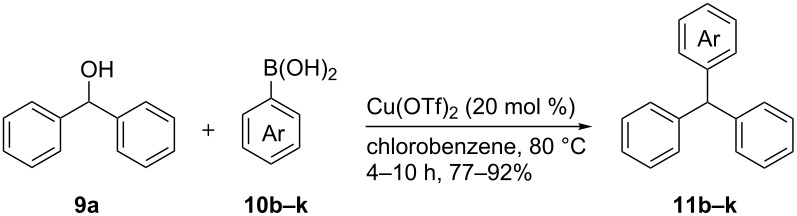				

Entry	Ar in arylboronic acid	Triarylmethane	Time (h)	Yield (%)

1	**10b**: 4-CF_3_C_6_H_4_	**11b**	4	92
2	**10c**: 4-FC_6_H_4_	**11c**	10	77
3	**10d**: 4-ClC_6_H_4_	**11d**	6	79
4	**10e**: 4-BrC_6_H_4_	**11e**	4	91
5	**10f**: 4-MeOC_6_H_4_	**11f**	5	81
6	**10g**: 4-MeC_6_H_4_	**11g**	6	81
7	**10h**: 2,5-(OMe)_2_C_6_H_3_	**11h**	6	88
8	**10i**: 2-furyl	**11i**	8	85
9	**10j**: 2-thiophenyl	**11j**	8	89
10	**10k**: 2-benzothiophenyl	**11k**	10	86

**Scheme 4:**
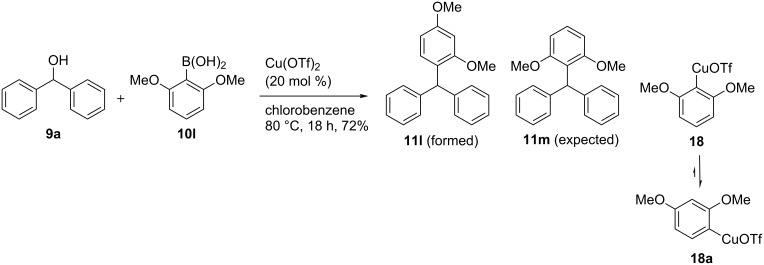
Copper-catalyzed C–C bond formation synthesis of triarylmethane **10l**.

However, when we employed 2,6-dimethoxyphenylboronic acid **10l**, surprisingly, we isolated the triarylmethane **11l**, in which the C–C bond formation took place on the C(3) carbon of the 2,6-dimethoxyphenylboronic acid instead of the C(1) carbon, as illustrated in **11m** (Scheme 4). Structure of **11l** was readily confirmed on the basis of ^13^C NMR and DEPT-135 spectra. We surmise that the initially formed, transmetallated product **18** rearranged to the more stable **18a** before it could react with diphenylmethanol (**9a**, Scheme 4).

The scope of the copper-catalyzed coupling reaction of diarylmethanols **9b–d** with phenylboronic acid (**10a**) was explored by changing one or both of the aryl rings in the diarylmethanol (Table 3) [62]. The copper-catalyzed reaction of phenyl(pyren-1-yl)methanol (**9b**) with phenylboronic acid (**10a**) was very facile and the product triarylmethane **11n** was obtained in 72% yield (Table 3, entry 1). Similarly, the reaction of anthracen-9-yl(phenyl)methanol (**9c**) with phenylboronic acid (**10a**) provided the corresponding triarylmethane **11o** in 82% yield (Table 3, entry 2). The last example in the genre is interesting, as one of the aryl rings is ferrocene in **11p**. The reaction of ferrocene-1-yl(phenyl)methanol (**9d**) with phenyboronic acid (**10a**) was facile and it provided diphenylmethylferrocene (**11p**) without any difficulty in 71% yield.

**Table 3:** Scope of diarylmethanol **9b–d** in the copper-catalyzed coupling reaction.

Entry	Diarylmethanol	Arylboronic acid	Triarylmethane^a^

1	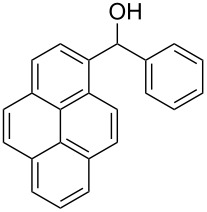 **9b**	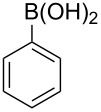 **10a**	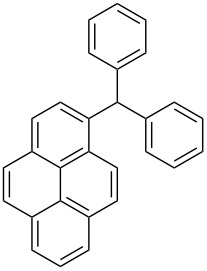 **11n** (14 h, 72%)
2	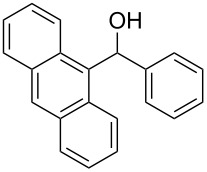 **9c**	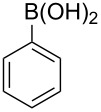 **10a**	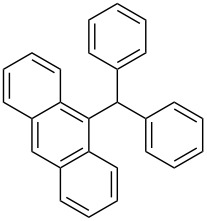 **11o** (16 h, 83%)
3	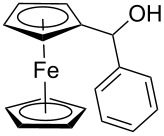 **9d**	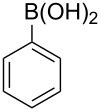 **10a**	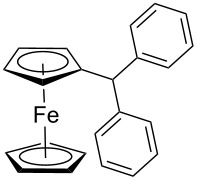 **11p** (16 h, 71%)
4	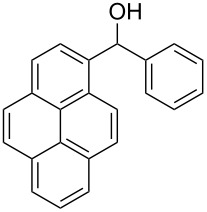 **9b**	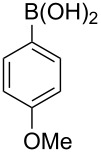 **10f**	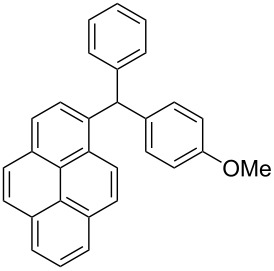 **11q** (16 h, 68%)
5	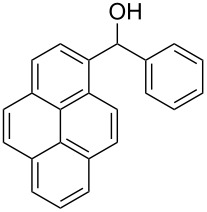 **9b**	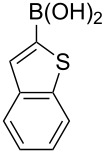 **10k**	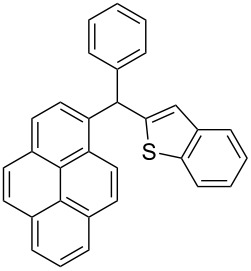 **11r** (12 h, 72%)
6	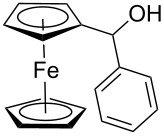 **9d**	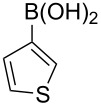 **10m**	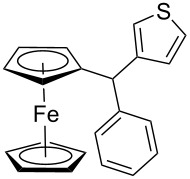 **11s** (4 h, 64%)

^a^Time required for completion of the reaction and yield of the isolated and purified triarylmethanes are given in the parenthesis.

### Modular synthesis of triarylmethanes

The synthetic method that we developed, through which three different aromatic rings on the central carbon can be assembled in a two-step protocol, is modular in nature. The first step is the synthesis of diarylmethanol and the second step is the replacement of the hydroxy group in the resulting diarylmethanol by a third aryl group by employing arylboronic acid under copper catalysis. As a proof of principle, we present the synthesis of three examples of triarylmethanes **11q–s** that bear three different aromatic rings (Table 3). The copper-catalyzed coupling reaction of phenyl(pyren-1-yl)methanol (**9b**) with 4-methoxyphenylboronic acid (**10f**) and benzo[*b*]thiophen-2-ylboronic acid (**10k**) provided the respective pyrene-containing unsymmetrical triarylmethanes **11q–r** in good yields (Table 3). Next, the coupling reaction of phenyl(ferrocenyl)methanol (**9d**) with thiophen-3-ylboronic acid (**10m**) provided triarylmethane **11s**, which has ferrocene, thiophene and phenyl rings installed on the central carbon. The triarylmethane **11s** was found to be unstable when kept as a solution in hexane. However, the compound was stable as a solid for at least two months when refrigerated (+5 °C).

**Scheme 5:**
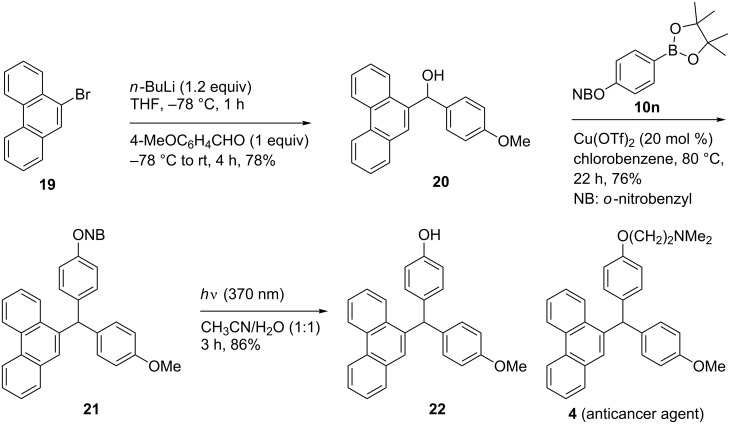
Synthesis of anti-breast-cancer agent intermediate **22**.

To demonstrate an application of our newly developed Cu(OTf)_2_-catalyzed C–C coupling reaction for the synthesis of triarylmethanes, we designed a synthesis of the precursor **22** (Scheme 5) for the anti-breast-cancer agent **4** (Figure 1). Any method for the synthesis of **4** needs to take into account that it has two phenyl rings with different alkoxy groups at the respective C(4) position. We reasoned that one of the aryl groups could be a part of diarylmethanol and the other of the arylboronic acid. We designed the protection of the C(4) hydroxy group in the arylboronic aicd with the photolabile 2-nitrobenzyl (NB) group, so that it can be removed without affecting the rest of the molecule. The synthesis of triarylmethane **22** began with the preparation of the starting diarylmethanol **20**, which was accomplished by the addition the anion from 9-bromophenanthrene [63] **19** to 4-methoxybenzaldehdye. The resulting diarylmethanol **20** was treated with bis(pinacolato)diboron [64] **10n**, which has an 2-nitrobenzyl protecting group on the phenolic hydroxy group [52]. The reaction was conducted in the presence of 20 mol % Cu(OTf)_2_ under optimized conditions, providing triarylmethane **21** in 76% yield. Deprotection of the phenolic hydroxy group in **21** was facile under photocatalytic conditions by using UV LED lamps in wet acetonitrile. The reaction furnished the synthetic intermediate **22** in 86% yield [52]. Since the intermediate **22** has been previously converted into the drug candidate **4** [49], our efforts constitute a formal, alternate synthesis.

## Conclusion

In conclusion, we have demonstrated a facile Cu(OTf)_2_-catalyzed synthesis of a variety of triarylmethanes from readily available diarylmethanols and arylboronic acids. This method is a novel synthetic approach for the preparation of multisubstituted triarylmethanes starting from easily preparable diarylmethanols and commercially available arylboronic acids. Structurally diverse, unsymmetrical triarylmethanes were prepared by employing this methodology. As an application to the newly developed methodology, we achieved a facile synthesis of the penultimate intermediate of an anti-breast-cancer agent. Hopefully the work described here will stimulate further work for the synthesis of a wide variety of triarylmethanes with tailor-made properties.

## Supporting Information

File 1 Experimental procedures, characterization data, details of the NMR structural determination of all new compounds and copies of ^1^H, ^13^C NMR and DEPT-135 spectra for all compounds prepared.
