# Supplementary material for: Copper-mediated arylation with arylboronic acids: Facile and modular synthesis of triarylmethanes
Source: Beilstein J Org Chem. 2016 Mar 11;12:496–504. doi: 10.3762/bjoc.12.49 (PMC4902010; doi:10.3762/bjoc.12.49)
Supplement: File 1 — Experimental procedures, characterization data, details of the NMR structural determination of all new compounds and copies of 1H, 13C NMR and DEPT-135 spectra for all compounds prepared. [file Beilstein_J_Org_Chem-12-496-s001.pdf]

Supporting Information

for

Copper-mediated arylation with arylboronic acids: Facile and modular synthesis of triarylmethanes

H. Surya Prakash Rao,* A. Veera Bhadra Rao

Address: Department of Chemistry, Pondicherry University, Pondicherry – 605 014. India,

Telephone: +914132654411; Fax: +914132656230 http://www.pondiuni.edu.in

E.mail: H. Surya Prakash Rao - hspr.che@pondiuni.edu.in;

*Corresponding author

Table of contents

General experimental section S2

General procedure for synthesis of triarylmethanes **11a–s** S2

Experimental details of compounds S3–S9

Reference S10

Copies of ^{1}H NMR and ^{13}C NMR and DEPT-135 spectra of compounds S11–S28

General experimental section

Progression of all the reactions was monitored by TLC using a hexanes (60–80 °C boiling mixture)/ethyl acetate mixture as eluent. Column chromatography was performed on silica gel (100–200 mesh) using increasing percentage of ethyl acetate in hexanes. ^{1}H NMR spectra (400 MHz), ^{13}C NMR (100MHz) and DEPT–135 spectra were recorded for ($CDCl_3$, $CDCl_3$ + CCl_4 (1:1)) solutions on a 400 MHz spectrometer with TMS as internal standard. Coupling constants *J* are given in Hz. IR spectra were recorded as KBr pellets on a FTIR spectrometer. High resolution mass spectra were recorded on quadrupole-time-of-flight (QTOF) mass spectrometer using electrospray ionization mode. Organic solvents were dried by standard methods. The catalyst $Pd_2(dba)_3$ and the alkenyl boronic acids were prepared according to literature procedures. Light-mediated deprotection of NB group to generate isoindolinones was carried out using home-built reactor having four UV-LED (3µW) lamps with an emission maximum at 370 nm.

General procedure for synthesis of triarylmethanes 11a–s.

Synthesis of triphenylmethane 11a: The procedure described here is analogous to our previously described method for the preparation of 3-substituted isoindolinones [52]. An oven-dried 25 mL two-neck round-bottom flask connected to Schlenk line through a condenser was charged with phenylboronic acid (**10a**, 130 mg, 1.08 mmol), $Cu(OTf)_2$ (77 mg, 0.21 mmol). The flask was sealed with a rubber septum, evacuated and purged with nitrogen gas three times. Anhydrous chlorobenzene (2 mL) was added through a syringe and the contents were stirred for 10 min. Diphenylmethanol (**9a**, 200 mg, 1.08 mmol) in chlorobenzene (4 mL) was next added over 5 min at rt (30 °C). The resulting reaction mixture was heated at 80 °C for 18 h while

periodically checking by TLC for completion of the reaction. The reaction mixture was then extracted with ethyl acetate (EtOAc, 2 × 10 mL). The organic layer was washed with water (2 × 20 mL), brine (1 × 10 mL) followed by removal of EtOAc under reduced pressure. The crude product was subjected to column chromatography (silica gel, gradient elution with increasing volume of 1% to 5% EtOAc in hexanes) to yield triphenylmethane (**11a**) as a colorless oil (220 mg, 78% yield).

Experimental details of compounds

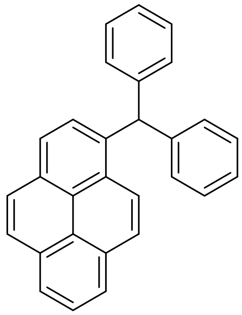

1-Benzhydrylpyrene (11n): Colorless liquid (221 mg, 72% yield); IR (KBr, cm^{-1}) 3040, 2922, 2853, 1642, 1451, 1076, 1026, 1260, 841, 743; ^1H NMR (400 MHz, $CDCl_3$ + CCl_4; 1:1) δ 8.23 (d, J = 9.3 Hz, 1H) 8.14-8.09 (m, 2H), 8.04 (d, J = 8.1 Hz, 1H), 8.00-7.93 (m, 4H), 7.50 (d, J = 8.0 Hz, 1H), 7.29-7.23 (m, 6H), 7.14-7.12 (m, 4H), 6.59 (s, 1H); ^{13}C NMR (100 Hz, $CDCl_3$ + CCl_4; 1:1) δ 144.3 (C), 137.8 (C), 131.6 (C), 130.9 (C), 130.3 (C), 130.0 (C), 129.2 (CH), 128.8 (C), 128.6 (CH), 127.9 (CH), 127.8 (CH), 127.6 (CH), 127.3 (CH), 126.6 (CH), 126.0 (CH), 125.38 (CH), 125.33 (C), 125.1 (CH), 124.7 (CH), 123.7 (CH), 53.5 (CH); HRMS (ESI) calcd for $C_{29}H_{21}$ (M + H) 369.1643, found 369.1646.

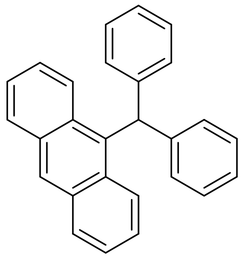

9-Benzhydrylanthracene (11o): Colorless solid (198 mg, 82% yield), Mp: 142 °C; IR (KBr, cm^{-1}) 2922, 2854, 1643, 1450, 1022, 785, 737, 701; ^1H NMR (400 MHz, $CDCl_3$ + CCl_4; 1:1) δ 8.43 (s, 1H), 8.12 (d, *J* = 8.8 Hz, 2H), 7.98 (d, *J* = 8.4 Hz, 2H), 7.38-7.34 (m, 2H), 7.27-7.16 (m, 12H), 7.07 (s, 1H); ^{13}C NMR (100 MHz, $CDCl_3$ + CCl_4; 1:1) δ 143.5 (C), 135.4 (C), 132.2 (C), 131.0 (C), 129.5 (CH), 129.3 (CH), 128.6 (CH), 128.1 (CH), 126.3 (CH), 126.0 (CH), 125.6 (CH), 124.8 (CH), 50.7 (CH); HRMS (ESI) calcd for $C_{27}H_{21}$ (M + H) 345.1643, found 345.1637.

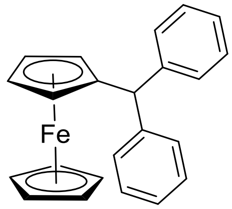

Diphenylmethyl ferrocene (11p): brown oil (149 mg, 71% yield),; IR (KBr, cm^{-1}) 3087, 3060, 3026, 1599, 1493, 1452, 1427, 1075, 1028 755, 703; ^1H NMR (400 MHz, $CDCl_3$ + CCl_4; 1:1) δ 7.25-7.21 (m, 4H), 7.16-7.12 (m, 6H), 5.11 (s, 1H), 4.11 (t, *J* = 8.0 Hz, 2H). 3.96-3.95 (m, 7H); ^{13}C NMR (100 MHz, $CDCl_3$ + CCl_4; 1:1) δ 145.2 (C), 129.0 (CH), 128.2 (CH), 126.3 (CH), 91.8 (C), 69.0 (CH), 68.9 (CH), 67.8 (CH), 52.1 (CH); HRMS (ESI) calcd for $C_{23}H_{20}Fe$ (M +) 352.0914, found 352.0906.

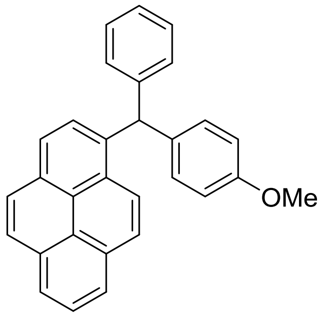

1-((4-Methoxyphenyl)(phenyl)methyl)pyrene (11q). Colorless solid (175 mg, 68% yield), Mp: 68 °C; IR (KBr, cm^{-1}) 3068, 2934, 2891, 1521, 1483, 1452, 1027, 813, 749, 733, 706; ^1H NMR (400 MHz, $CDCl_3$ + CCl_4; 1:1) δ 8.21 (d, *J* = 9.6 Hz, 1H), 8.10-7.89 (m, 8H), 7.49 (d, *J* = 8.0 Hz, 1H), 7.25-7.23 (m, 2H), 7.11 (d, *J* = 7.2 Hz, 2H), 7.01 (d, *J* = 8.4 Hz, 2H), 6.78 (d, *J* = 8.4 Hz, 2H), 6.52 (s, 1H), 3.73 (s, 3H); ^{13}C NMR (100 Hz, $CDCl_3$ + CCl_4; 1:1) δ 158.3 (C), 144.6 (C), 138.1 (C), 136.3 (C), 131.6 (C), 130.96 (C), 130.90 (C), 130.3 (CH), 129.6 (CH), 129.1 (C), 128.68 (C), 128.63 (CH), 127.8 (C), 127.75 (CH), 127.70 (CH), 127.2 (CH), 126.5 (CH), 126.0 (CH), 125.3 (CH), 125.2 (CH), 125.1 (CH), 124.7 (CH), 123.8 (CH), 114.0 (CH), 55.2 (CH_3), 52.7 (CH); HRMS (ESI) calcd for $C_{30}H_{23}O$ (M + H) 399.1743, found 399.1710.

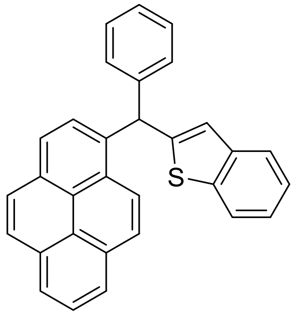

2-(Phenyl(pyren-1-yl)methyl)benzo[*b*]thiophene (11r). Colorless liquid (198 mg, 72% yield); IR (KBr, cm^{-1}) 3045, 2926, 2872, 1600, 1492, 1449, 1077, 800, 750, 733, 700; ^1H NMR (400 MHz, $CDCl_3$ + CCl_4; 1:1) δ 8.27 (d, *J* = 9.3 Hz, 1H), 8.14-8.10 (m, 2H), 8.02-7.95 (m, 5H), 7.84 (d, *J* = 8.0 Hz, 2H), 7.54 (d, *J* = 8.0 Hz, 1H), 7.38 (d, *J* = 8.0 Hz, 1H), 7.28-7.22 (m, 6H), 7.38 (t, *J* = 7.4 Hz, 1H), 6.77 (s, 1H), 6.66 (s, 1H); ^{13}C NMR (100 Hz, $CDCl_3$ + CCl_4; 1:1) δ 142.9 (C),

141.0 (C), 139.6 (C), 138.6 (C), 136.3 (C), 131.6 (C), 130.9 (C), 130.6 (C), 129.6 (2 × CH), 129.0 (C), 128.8 (CH), 128.1 (CH), 127.7 (CH), 127.3 (CH), 127.1 (CH), 127.0 (CH), 126.1 (CH), 125.5 (C), 125.4 (CH), 125.2 (CH), 125.1 (C), 124.9 (CH), 124.5 (CH), 124.2 (CH), 123.3 (CH), 123.0 (CH), 122.9 (CH), 48.0 (CH); HRMS (ESI) calcd for $C_{31}H_{21}S$ (M + H) 425.1358, found 425.1359.

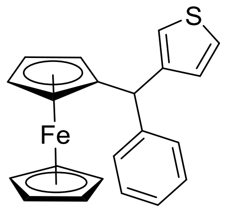

3-(Phenyl(ferrocenyl)methyl)thiophene (11s). brown oil (156 mg, 64% yield); IR (KBr, cm^{-1}); 3028, 1624, 1597, 1487, 1458, 1431, 1203, 1071, 1021 751, 707; ^{1}H NMR (400 MHz, $CDCl_3$ + CCl_4; 1:1) δ 7.26-7.24 (m, 2H), 7.19-7.15 (m, 4H), 6.83-6.82 (m, 2H), 5.12 (s, 1H), 4.11-4.04 (m, 3H), 3.96 (s, 5H) 3.93 (s, 1H); ^{13}C NMR (100 Hz, $CDCl_3$ + CCl_4; 1:1) δ 146.2 (C), 144.7 (C), 128.7 (C), 128.5 (C), 128.3 (C), 126.5 (C), 125.1 (C), 121.6 (C), 91.9 (C), 68.9 (CH), 68.4 (2 × CH), 67.8 (CH), 67.6 (CH), 47.6 (CH); HRMS (ESI) calcd for $C_{21}H_{18}FeS$ (M +) 358.0479, found 358.0472.

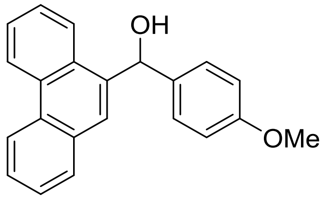

(4-Methoxyphenyl)(phenanthren-9-yl)methanol (20): An oven-dried 25 mL two neck RB connected with Schlenk line charged with 9-bromo phenathrene (500 mg, 1.94 mmol) in 10 mL THF cooled to −78 °C. To this *n*-BuLi (1.2 mL) was added dropwise over a time 10 min the rection mixture turned red; to this 4-methoxybenzaldehyde (208 mg, 1.94 mmol) in 5 mL THF was added slowly. The reaction m ixture was stirred for 4 h. The completion of the reaction was

monitored by TLC. The reaction mixture was quenched with saturated ammonium chloride solution and extracted with (DCM, 2 × 20 mL). The organic layer was washed with water (2 × 20 mL), brine (1 × 10 mL) followed by removal of the DCM under reduced pressure. The crude product was subjected to column chromatography (silica gel, gradient elution with increasing volume of EtOAc in hexanes) to yield (4-methoxyphenyl)(phenanthren-9-yl)methanol (**20**) as viscous solid (475 mg, 78% yield). IR (KBr, cm^{-1}); 3028, 1624, 1597, 1487, 1458, 1431, 1203, 1071, 1021 751, 707; ^{1}H NMR (400 MHz, $CDCl_3$ + CCl_4; 1:1) δ 8.71 (d, *J* = 8.4 Hz, 1H), 8.65 (d, *J* = 8.0 Hz, 1H), 7.98 (s, 1H), 7.95-7.89 (m, 2H), 7.65-7.48 (m, 4H), 7.33 (d, *J* = 8.4 Hz, 2H), 6.84-6.82 (m, 2H), 6.46 (s, 1H), 3.65 (s, 3H), 2.30 (bs, 1H); ^{13}C NMR (100 Hz, $CDCl_3$ + CCl_4; 1:1) δ 159.2 (C), 137.1 (C), 135.2 (C), 131.5 (C), 130.9 (C), 130.4 (C), 129.8 (C), 129.1 (CH), 128.7 (CH), 126.8 (2 × CH), 126.5 (CH), 126.2 (CH), 125.2 (CH), 125.0 (CH), 123.2 (CH), 122.5 (CH), 114.0 (CH), 73.5 (CH), 55.1 (CH_3); HRMS (ESI) calcd for $C_{22}H_{19}O_2$ (M + H) 315.1385, found 315.1397.

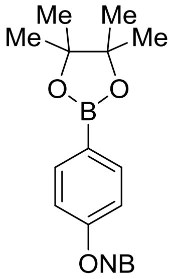

Synthesis of 4,4,5,5-tetramethyl-2-(4-((2-nitrobenzyl)oxy)phenyl)-1,3,2-dioxaborolane (10m): The procedure described here is analogous to our previously described method for preparation of dioxaborolane [1]. A viscous solution of 1-((4-bromophenoxy)methyl)-2-nitrobenzene (200 mg 0.65 mmol), bis(pinacolato)diboron (200 mg, 0.78 mmol), NaOAc (210 mg, 2.6 mmol) and $Pd(PPh_3)_2Cl_2$ (23 mg, 0.03 mmol) in PEG 400 (3 mL) was stirred in an oil bath at 80 °C for an 4 h under an atmosphere of nitrogen. After completion (TLC, 10% EtOAc in

hexanes, R_f = 0.4), the cooled reaction mixture was extracted with methyl *tert*-butyl ether (MTBE, 2 ×10 mL). The ether layer was washed with brine and dried over anhydrous sodium sulfate. Crude product obtained after removal of MTBE was purified by column chromatography (silica gel, gradient elution with increasing volume of EtOAc in hexanes) resulted **10m** as colourless viscous solid (215 mg, 93% yield), IR (KBr, cm^{-1}) 2948, 2839, 1605, 1508, 1455, 1251, 1172, 1028, 827, 678; ^1H NMR (400 MHz, $CDCl_3$ + CCl_4; 1:1) δ 8.16 (d, *J* = 8.4 Hz, 1H), 7.88 (d, *J* = 8.0 Hz, 1H), 7.76 (d, *J* = 8.8 Hz, 2H), 7.67-7.63 (m, 1H), 7.49-7.45 (m, 1H), 6.96 (d, *J* = 8.8 Hz, 2H), 5.51 (s, 2H), 1.33 (s, 12H); ^{13}C NMR (100 MHz, $CDCl_3$ + CCl_4; 1:1) δ 160.7 (C), 147.0 (C), 136.8 (CH), 134.9 (C), 134.0 (CH), 128.6 (CH), 128.3 (CH), 127.7 (C), 125. (CH), 114.2 (CH), 83.6 (C), 66.5 (CH_2), 25.0 (CH_3).

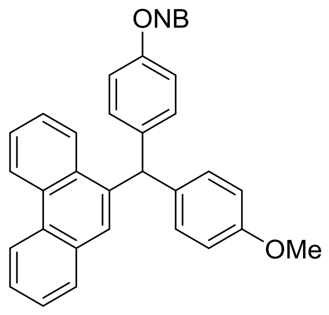

9-((4-Methoxyphenyl)(4-((2-nitrobenzyl)oxy)phenyl)methyl)phenanthrene (21): following the general procedure hydroxy compound **20** (100 mg, 0.31 mmo), 4,4,5,5-tetramethyl-2-(4-((2-nitrobenzyl)oxy)phenyl)-1,3,2-dioxaborolane (112 mg, 0.31 mmol) and $Cu(OTf)_2$ (11.4 mg, 0.062 mmol) in chlorobenzene resulted 9-((4-methoxyphenyl)(4-((2-nitrobenzyl)oxy)phenyl)methyl)phenanthrene **21** as viscous solid (126 mg, 76%). IR (KBr, cm^{-1}); 2839, 1733, 1653, 1604, 1513, 1452, 1249, 1170, 1031; ^1H NMR (400 MHz, $CDCl_3$ + CCl_4; 1:1) δ 8.72 (d, *J* = 8.4 Hz, 1H), 8.64 (d, *J* = 8.4 Hz, 1H), 8.16 (d, *J* = 8.4 Hz, 1H), 8.02 (d, *J* = 8.2 Hz, 1H), 7.93 (d, *J* = 8.0 Hz, 1H), 7.69-7.65 (m, 2H), 7.61-7.57 (m, 2H), 7.53-7.44 (m, 3H), 7.14 (s, 1H), 7.10-7.05 (m, 4H), 6.92 (d, *J* = 8.2 Hz, 2H), 6.82 (d, *J* = 8.4 Hz, 2H), 6.15 (s,

1H), 5.47 (s, 2H), 3.79 (s, 3H); ^{13}C NMR (100 MHz, $CDCl_3$ + CCl_4; 1:1) δ 158.2 (C), 156.8 (C), 147.0 (C), 138.8 (C), 137.0 (C), 135.8 (C), 134.3 (C), 134.0 (C), 131.6 (C), 131.3 (C), 131.0 (CH), 130.9 (CH), 130.7 (CH), 130.0 (CH), 128.9 (CH), 128.6 (CH), 128.5 (CH), 128.2 (CH), 126.7 (CH), 126.6 (CH), 126.5 (CH), 126.2 (CH), 125.4 (CH), 125.0 (CH), 123.2 (CH), 122.4 (CH), 115.0 (CH), 114.0 (CH), 67.0 (CH), 55.2 (CH_3), 52.0 (CH); HRMS (ESI) calcd for $C_{35}H_{27}NO_4Na$ (M + Na) 548.1832, found 548.1824.

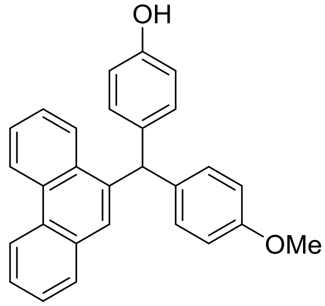

4-((4-Methoxyphenyl)(phenanthren-9-yl)methyl)phenol 22: The procedure is analogous to the one described for the 2-NB deprotection previously described by us [1]. The stirred solution of 9-((4-methoxyphenyl)(4-((2-nitrobenzyl)oxy)phenyl)methyl)phenanthrene (**21**, 50 mg, mmol) in CH_3CN/H_2O (1:1; 5 mL) in a Pyrex test tube of 20 mL capacity was exposed to light irradiation with emission maximum at 370 nm emitted by UV-LEDs (4 × 3µW) lamps. After completion of the deprotection (3 h) the solvent was removed under reduced pressure, and the resulting crude product was subjected to column chromatography (silica gel, gradient elution with increasing volume of EtOAc in hexanes) to separate the 4-((4-methoxyphenyl)(phenanthren-9-yl)methyl)phenol (**22**) from nitroso benzaldehyde. Compound **22** was obtained as viscous solid (31 mg, 86% yield). IR (KBr, cm^{-1}) 2929, 1731, 1609, 1509, 1451, 1248, 1032, 837, 750; ^{1}H NMR (400 MHz, $CDCl_3$ + CCl_4; 1:1) δ 8.71 (d, *J* = 8.0 Hz, 1H), 8.64 (d, *J* = 8.4 Hz, 1H), 8.01 (d, *J* = 8.3 Hz, 1H), 7.67 (d, *J* = 7.9 Hz, 1H), 7.58 (t, *J* = 7.3 Hz, 2H), 7.52-7.45 (m, 2H), 7.13 (s, 1H), 7.05 (d, *J* = 8.5 Hz, 2H), 6.99 (d, *J* = 8.6 Hz, 2H), 6.81 (d, *J* = 8.6 Hz, 2H), 6.73 (d, *J* = 8.3

Hz, 2H), 6.12 (s, 1H), 3.79 (s, 3H); ^{13}C NMR (100 MHz, $CDCl_3$ + CCl_4; 1:1) δ 158.2 (C), 154.3 (C), 139.0 (C), 136.14 (C), 136.11 (C), 131.6 (C), 131.4 (C), 131.0 (C), 130.9 (CH), 130.7 (CH), 130.0 (CH), 128.9 (CH), 128.5 (CH), 126.7 (CH), 126.6 (CH), 126.5 (CH), 126.2 (CH), 125.4 (CH), 123.2 (CH), 122.5 (CH), 115.5 (CH), 114.0 (CH), 55.2 (CH_3), 52.0 (CH); HRMS (ESI) calcd for $C_{28}H_{23}O_2$ (M + H) 391.1693, found 391.1693.

Reference

1. Rao, H. S. P.; Rao, A. V. B. J. Org. Chem. 2015, 80, 1506–1516. doi:10.1021/jo502446k

Copies of ^{1}H NMR and ^{13}C NMR and DEPT-135 spectra of compounds

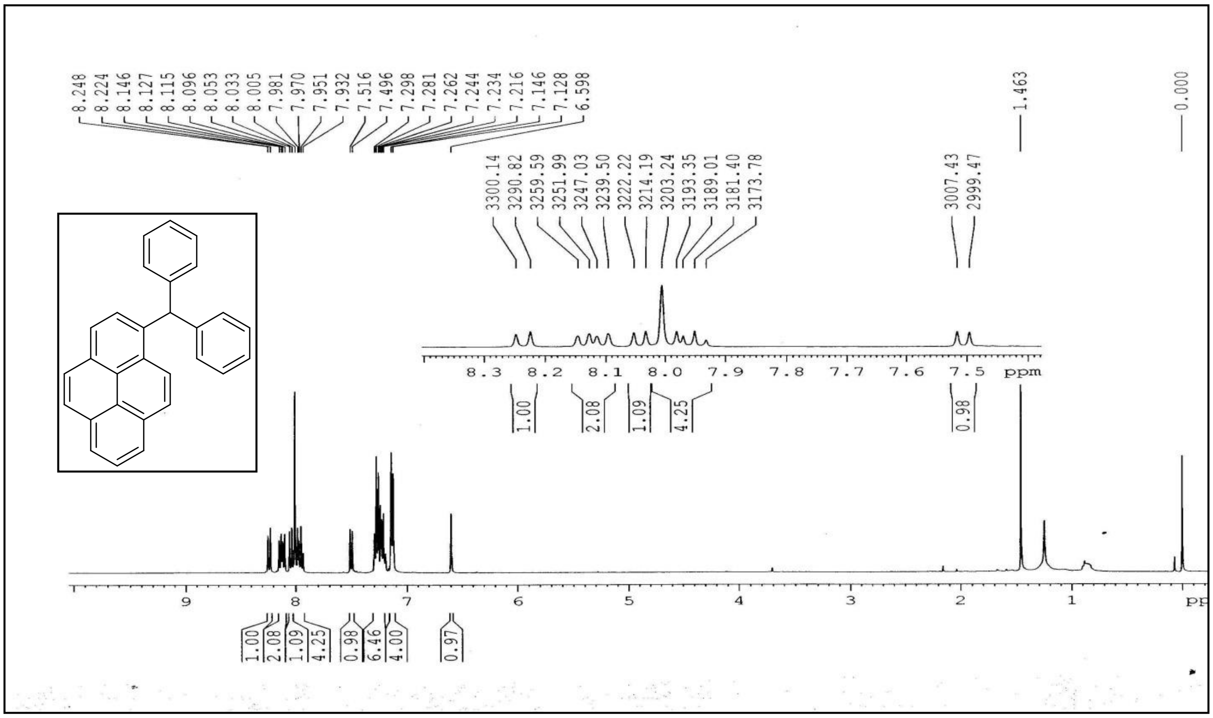

^{1}H NMR (400 MHz, $CDCl_3$ + CCl_4; 1:1) spectrum of 1-benzhydrylpyrene (**11n**).

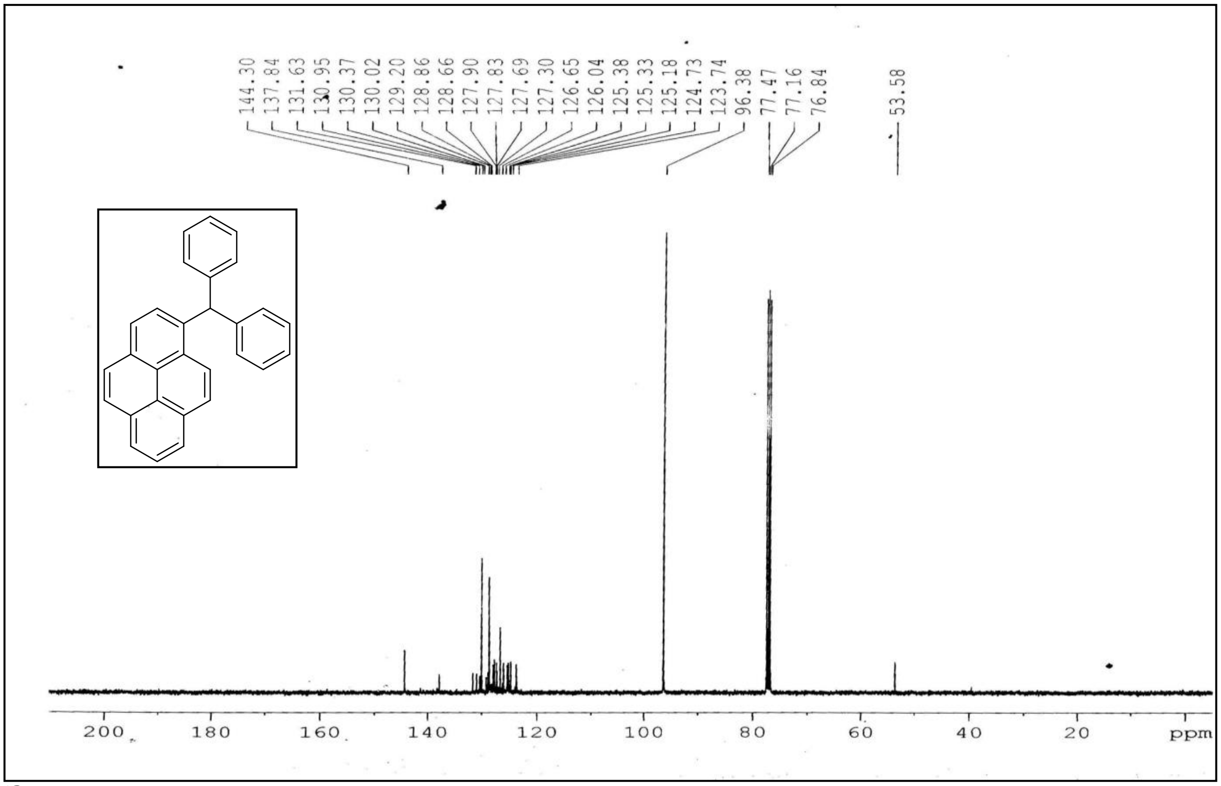

^{13}C NMR (100 MHz, $CDCl_3$ + CCl_4; 1:1) spectrum of 1-benzhydrylpyrene (**11n**).

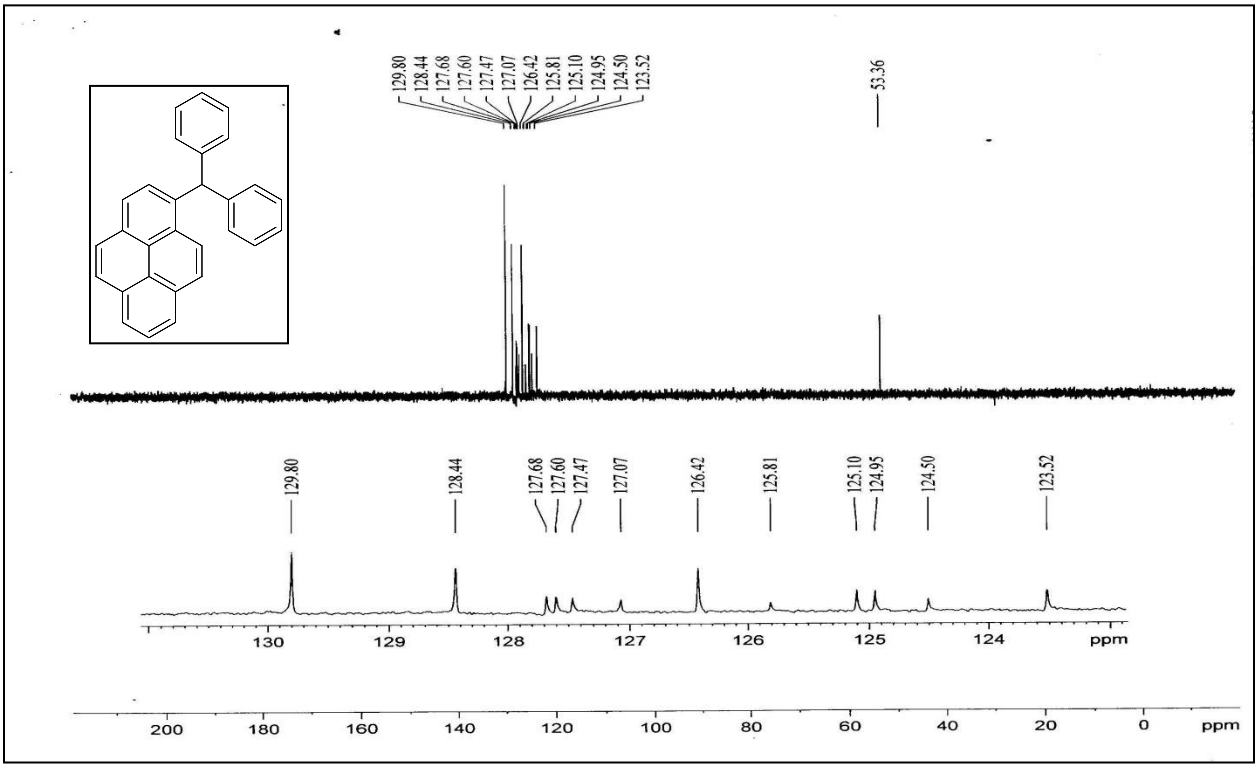

DEPT-135 spectrum of 1-benzhydrylpyrene (**11n**).

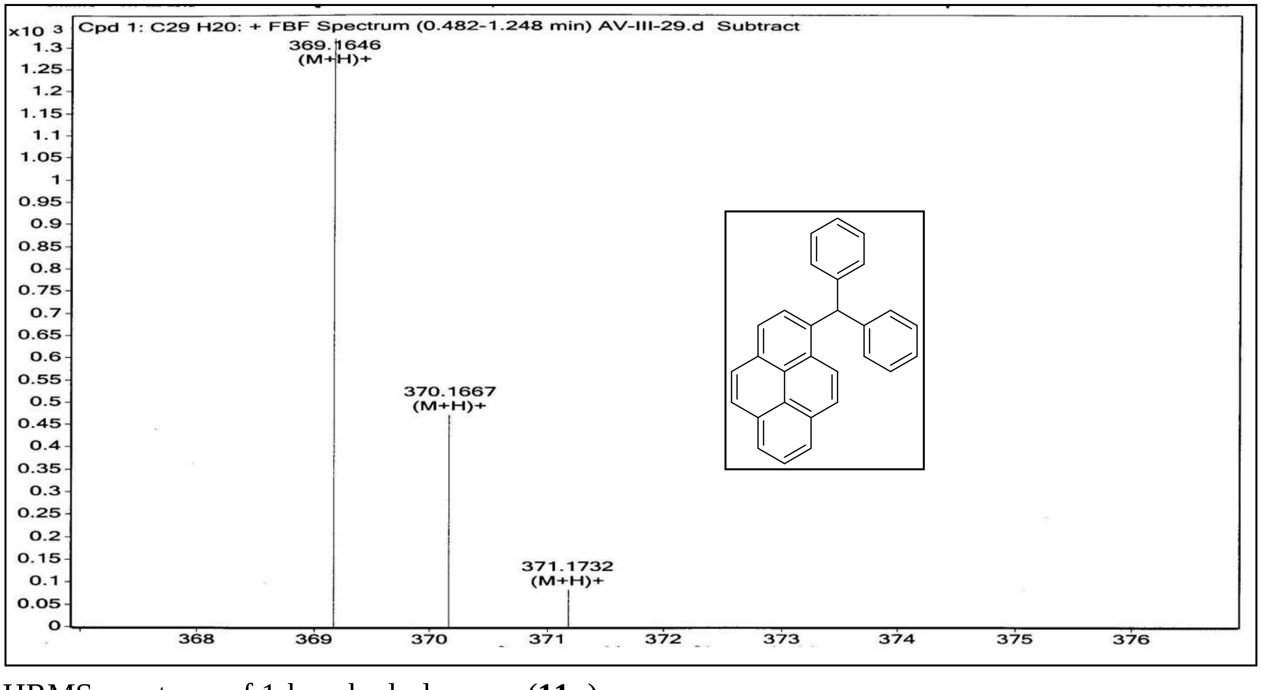

HRMS spectrum of 1-benzhydrylpyrene (**11n**).

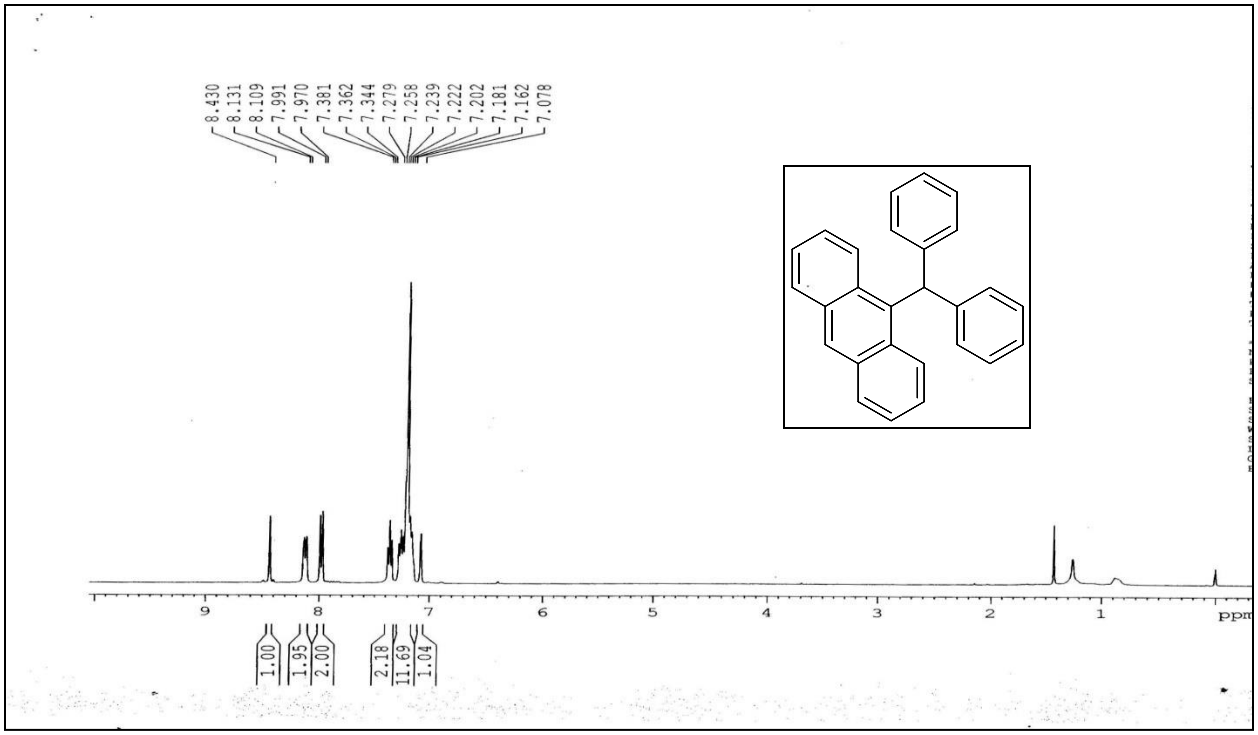

^{1}H NMR (400 MHz, $CDCl_3$ + CCl_4; 1:1) spectrum of 9-benzhydrylanthracene (**11o**)

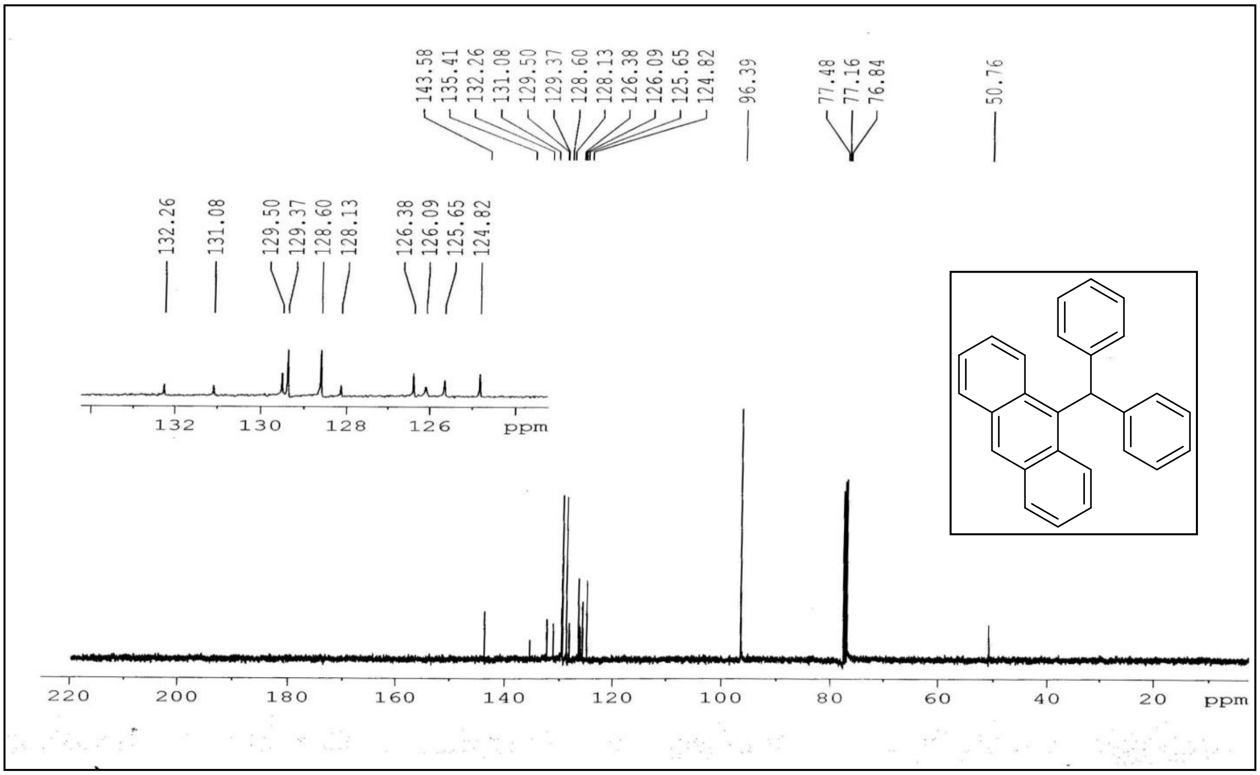

^{13}C NMR (100 MHz, $CDCl_3$ + CCl_4; 1:1) spectrum of 9-benzhydrylanthracene (**11o**).

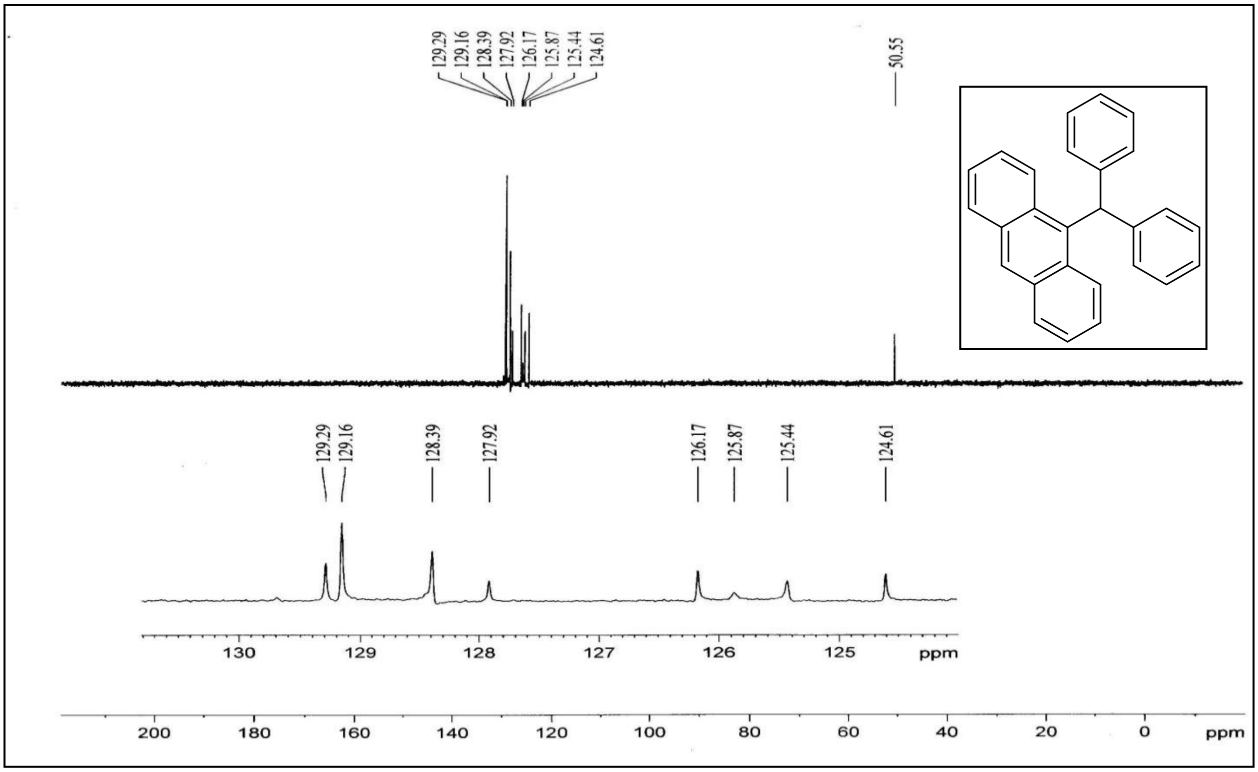

DEPT-135 spectrum of 9-benzhydrylanthracene (**11o**).

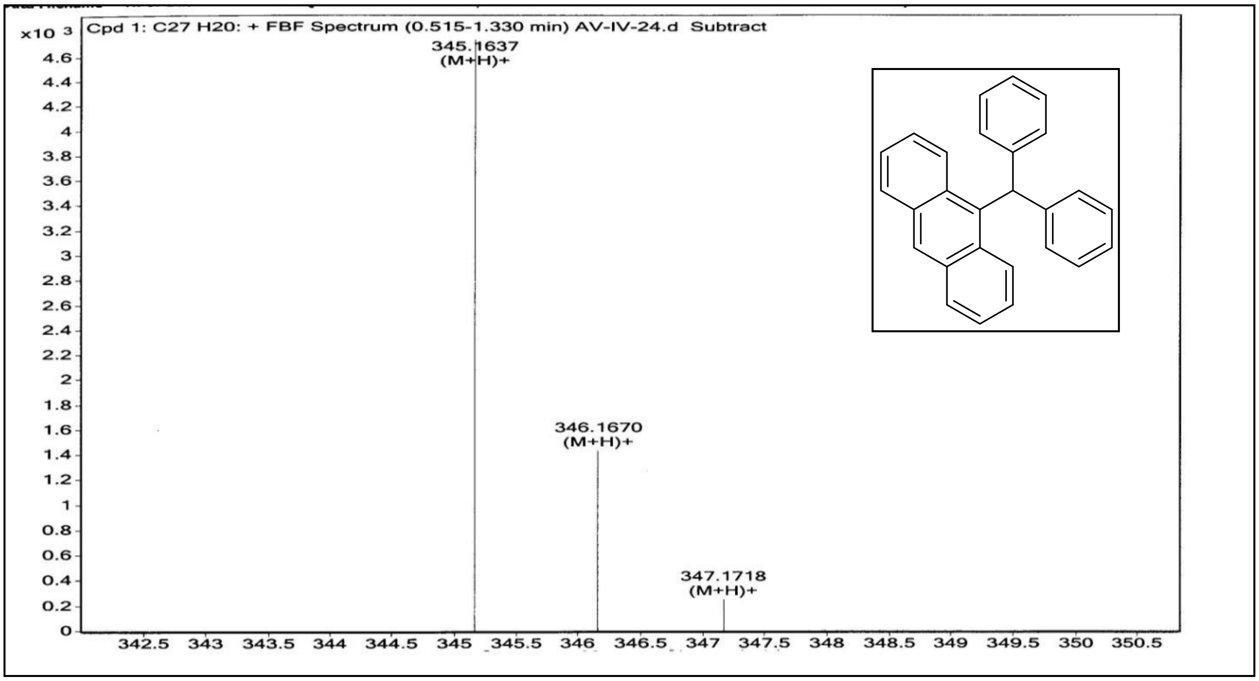

HRMS spectrum of 9-benzhydrylanthracene (**11o**).

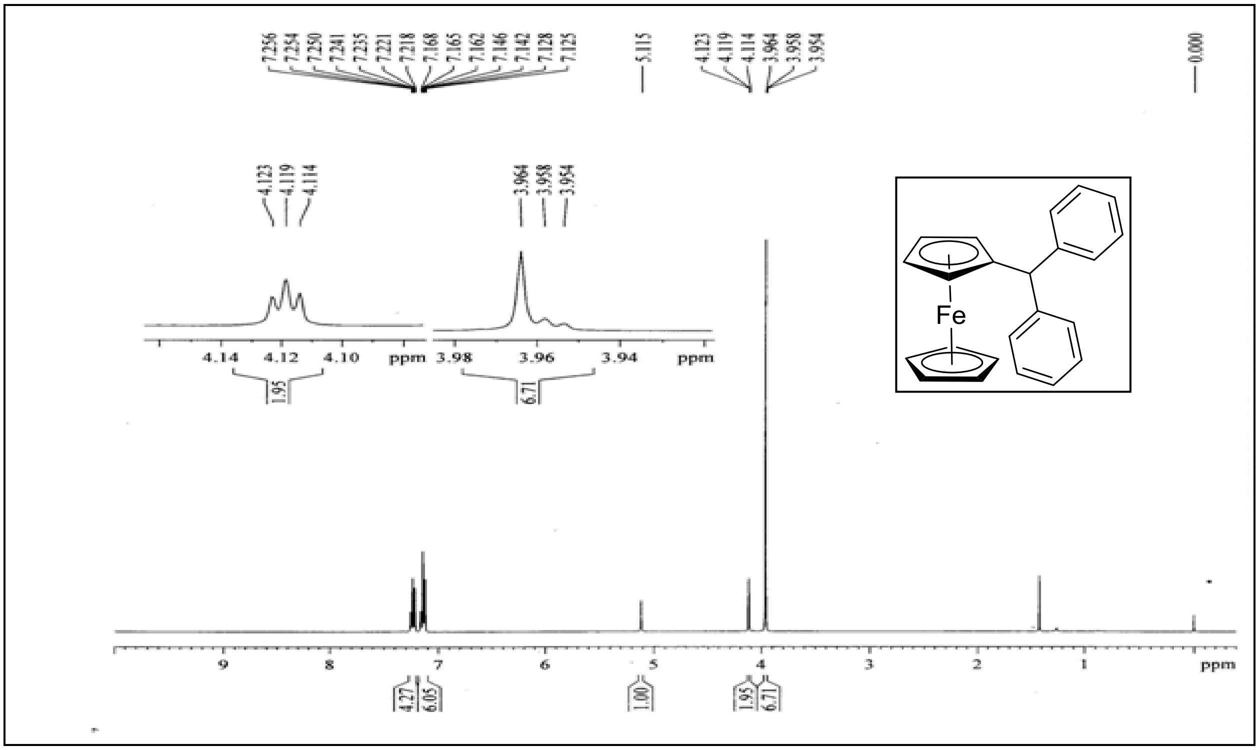

^{1}H NMR (400 MHz, $CDCl_3$ + CCl_4; 1:1) spectrum of diphenylmethyl ferrocene (**11p**).

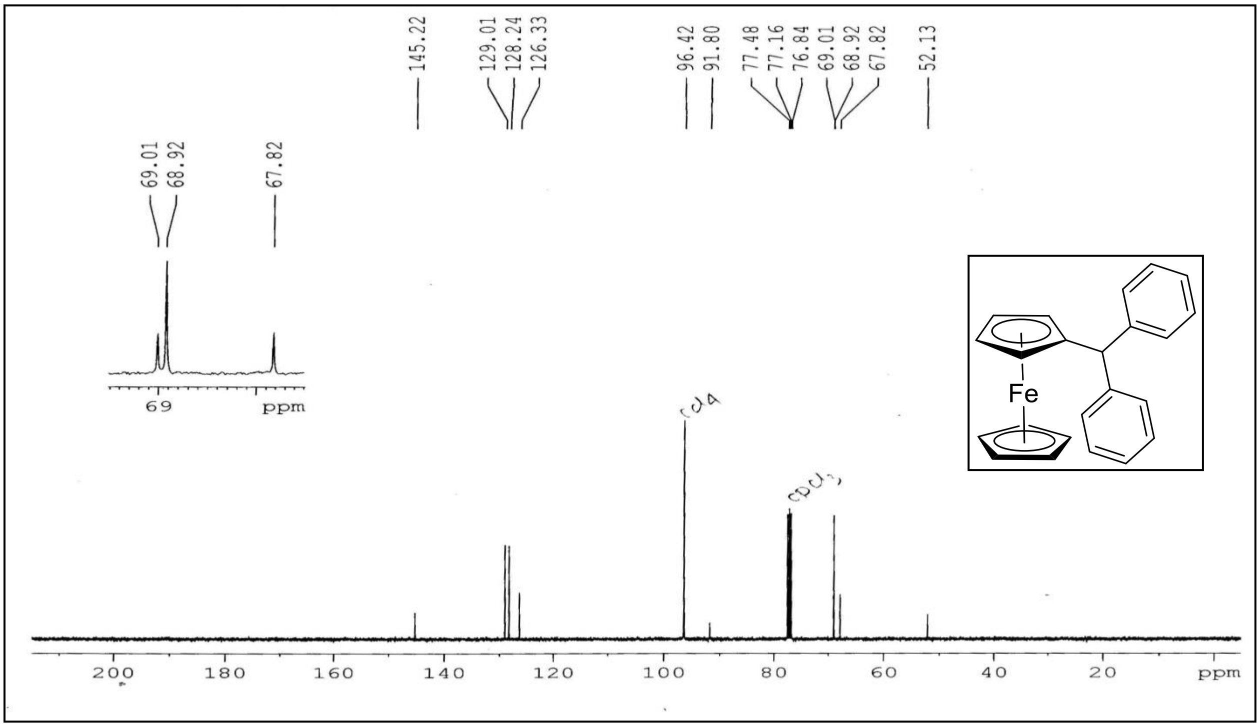

^{13}C NMR (100 MHz, $CDCl_3$ + CCl_4; 1:1) spectrum of diphenylmethyl ferrocene (**11p**).

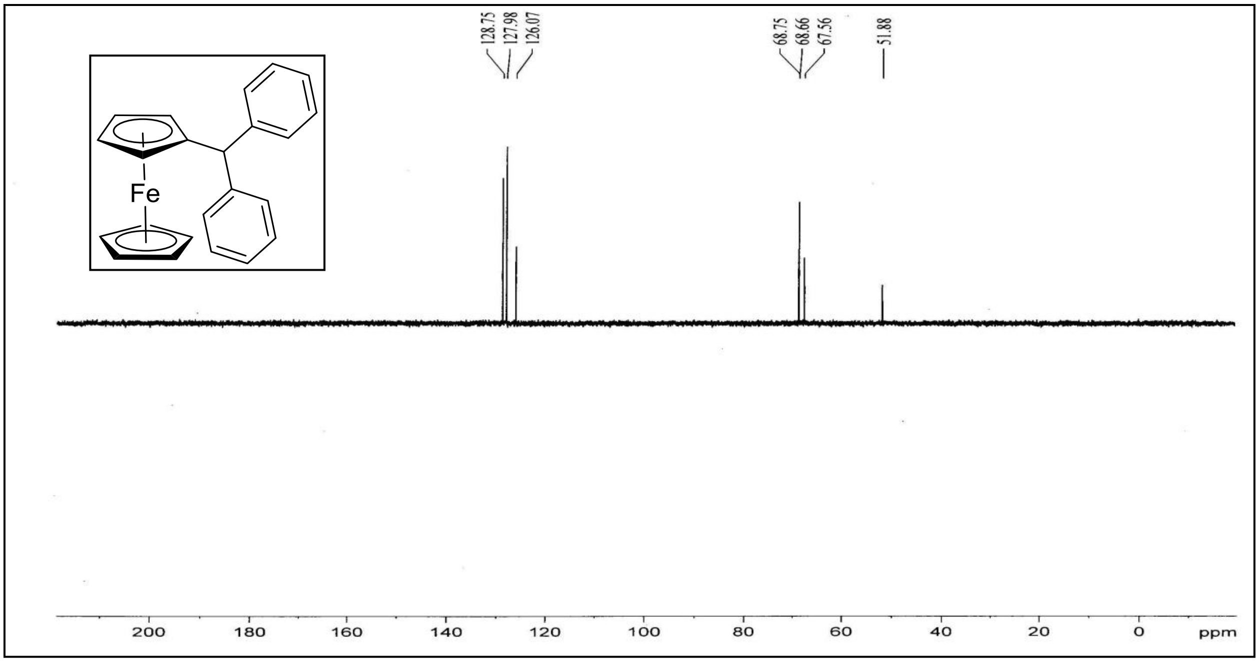

DEPT-135 spectrum of diphenylmethyl ferrocene (**11p**).

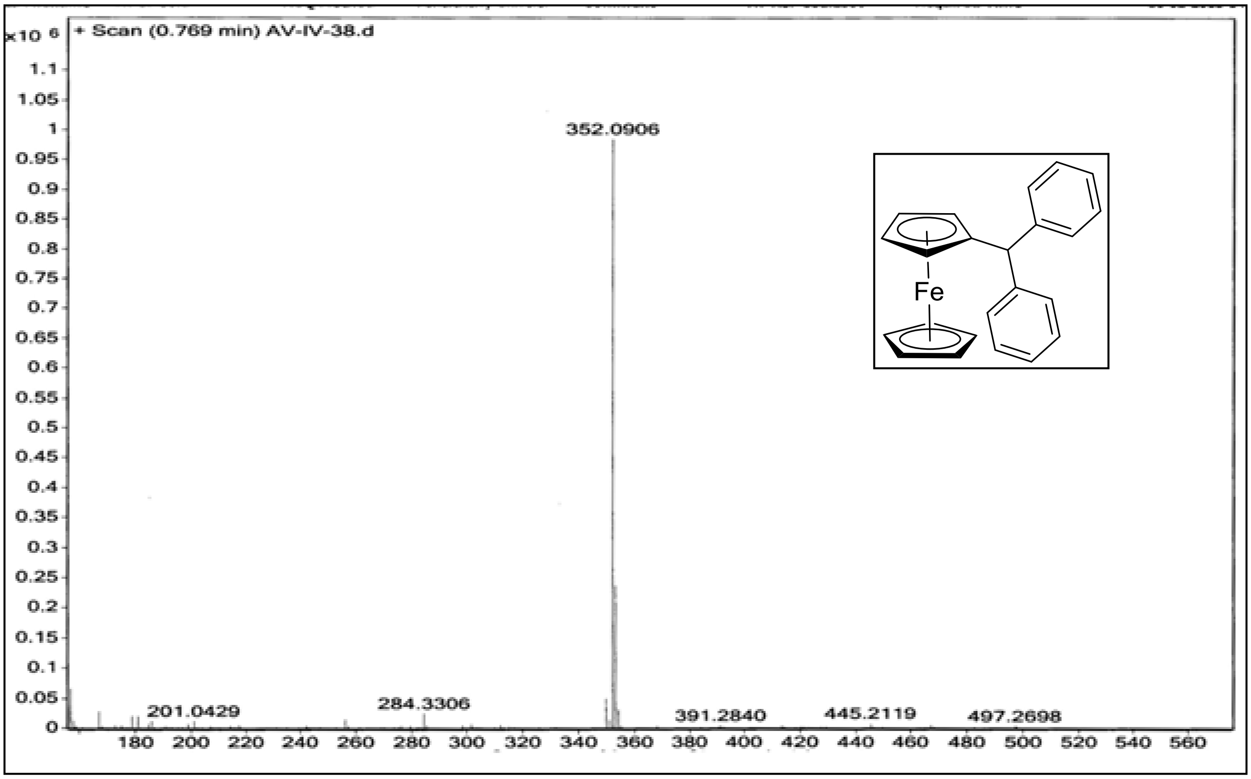

HRMS spectrum of diphenylmethyl ferrocene (**11p**).

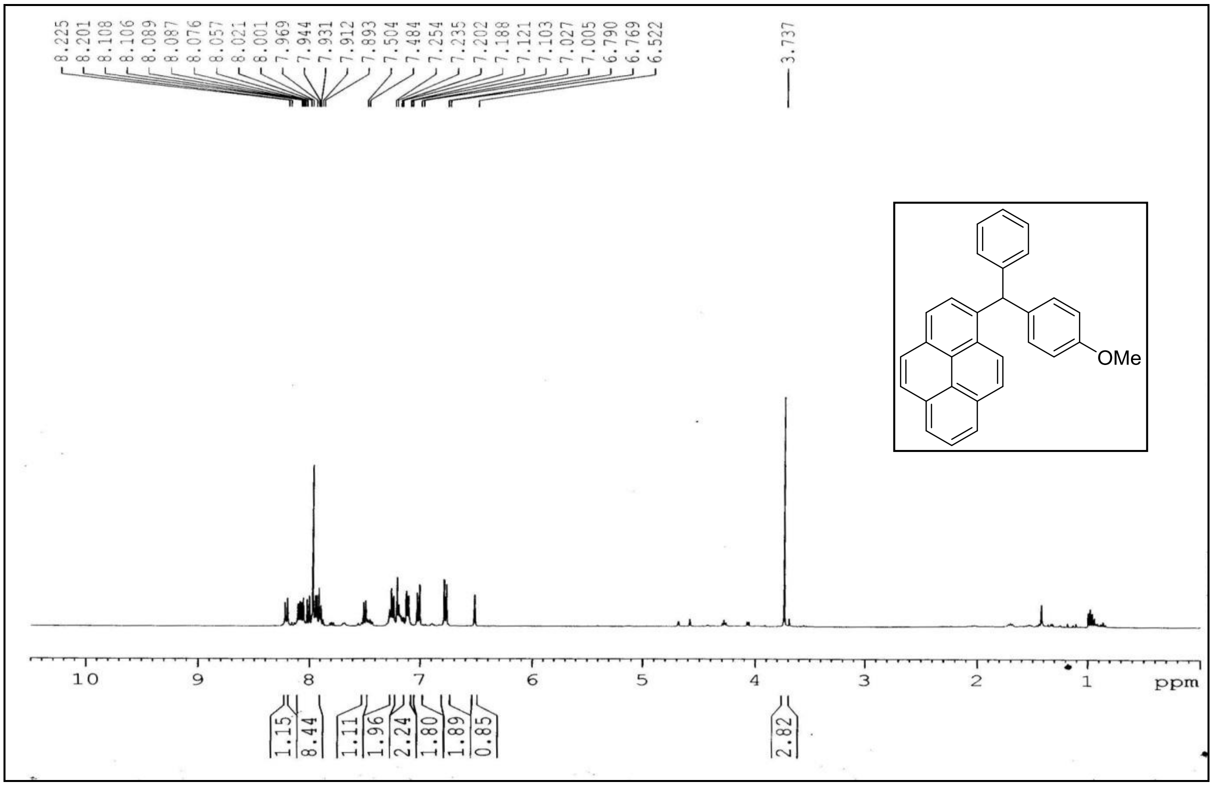

^{1}H NMR (400 MHz, $CDCl_3$ + CCl_4; 1:1) spectrum of 1-((4-methoxyphenyl)(phenyl)methyl)pyrene (**11q**).

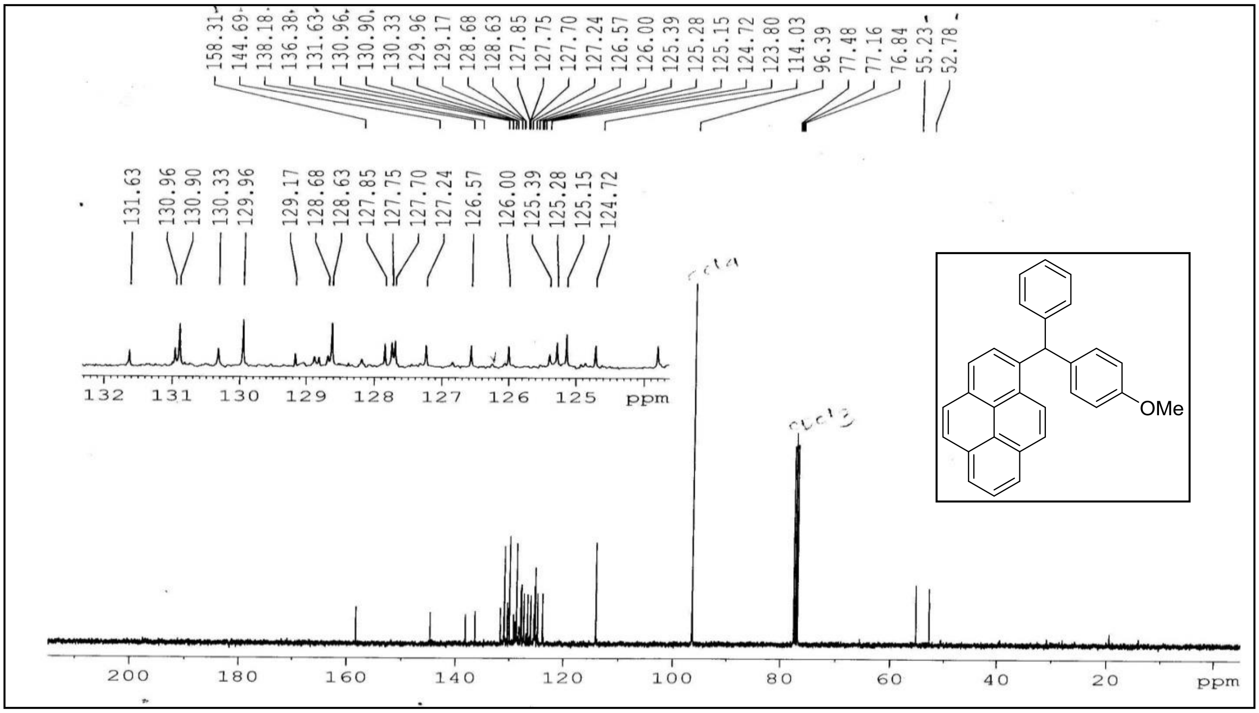

^{13}C NMR (100 MHz, $CDCl_3$ + CCl_4; 1:1) spectrum of 1-((4-methoxyphenyl)(phenyl)methyl)pyrene (**11q**).

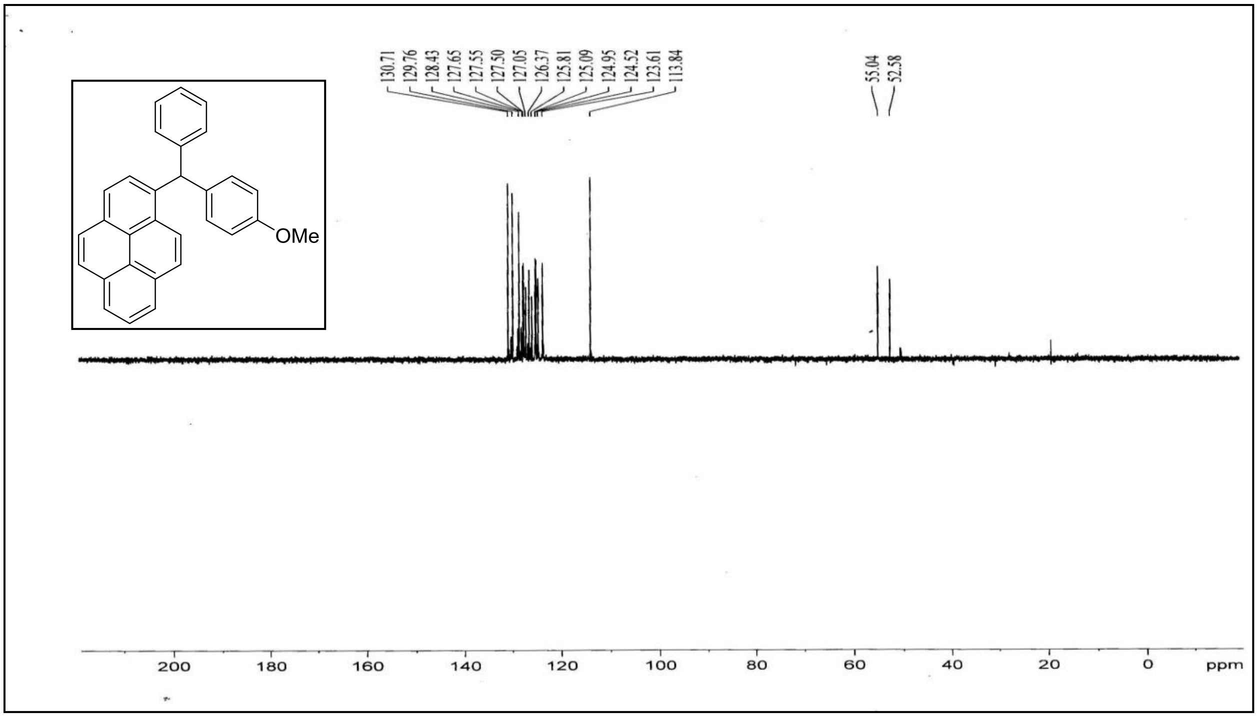

DEPT-135 spectrum of 1-((4-methoxyphenyl)(phenyl)methyl)pyrene (**11q**).

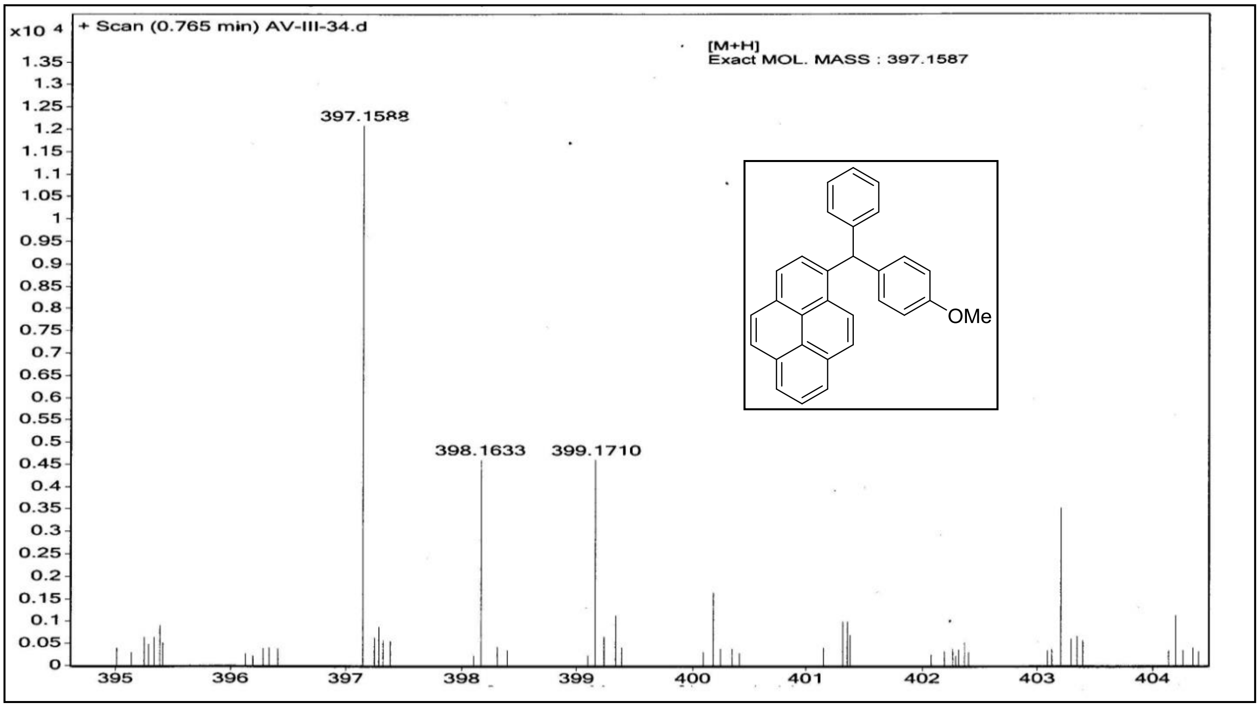

HRMS spectrum of 1-((4-methoxyphenyl)(phenyl)methyl)pyrene (**11q**).

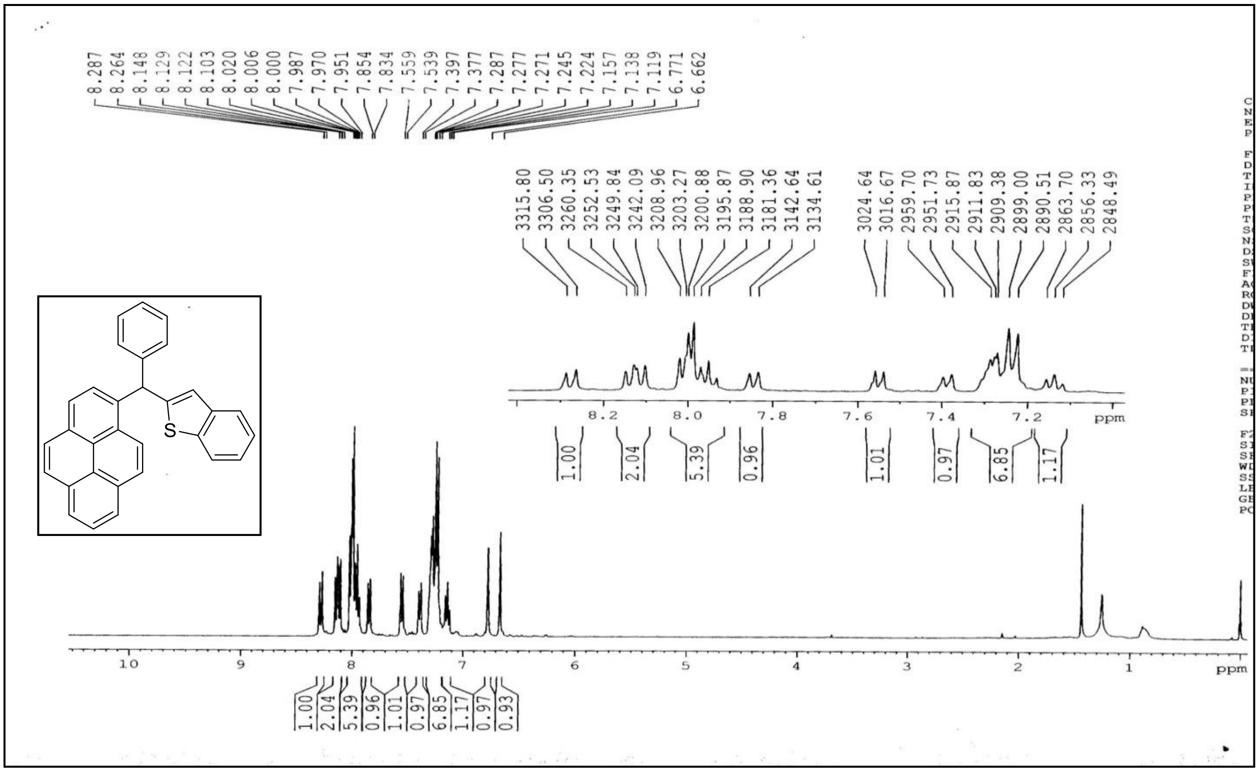

^{1}H NMR (400 MHz, $CDCl_3$ + CCl_4; 1:1) spectrum of 2-(phenyl(pyren-1-yl)methyl)benzo[*b*]thiophene (**11r**).

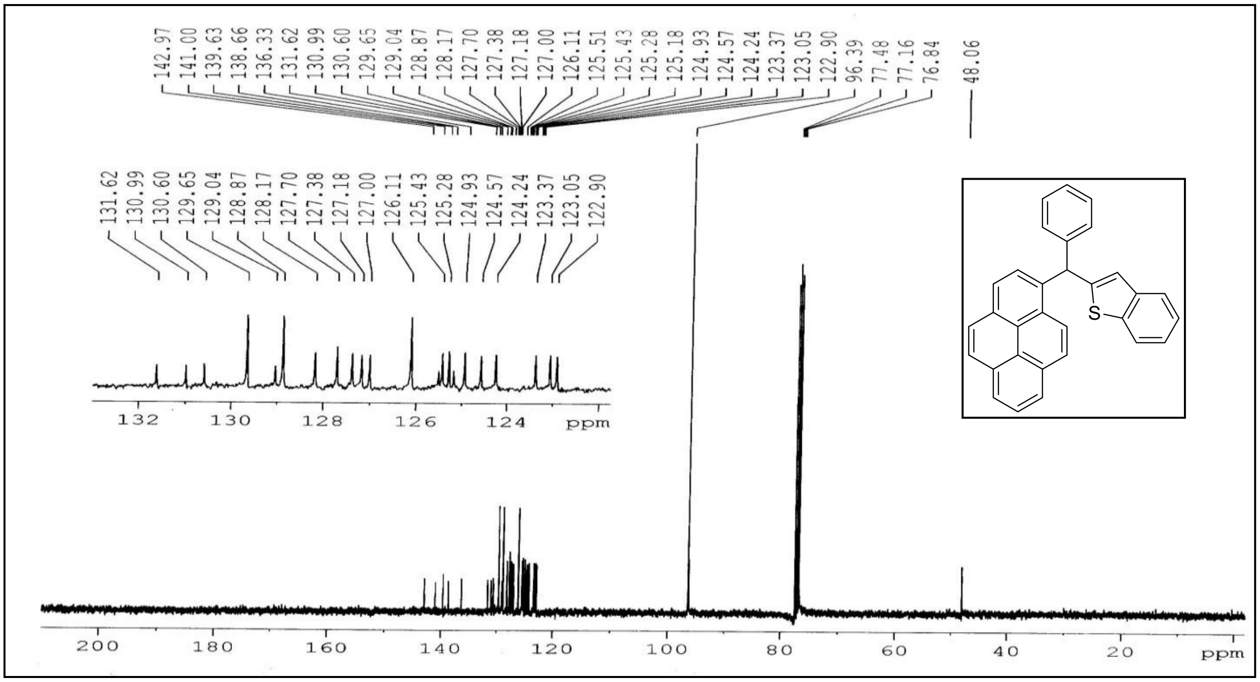

^{13}C NMR (100 MHz, $CDCl_3$ + CCl_4; 1:1) spectrum of 2-(phenyl(pyren-1-yl)methyl)benzo[*b*]thiophene (**11r**).

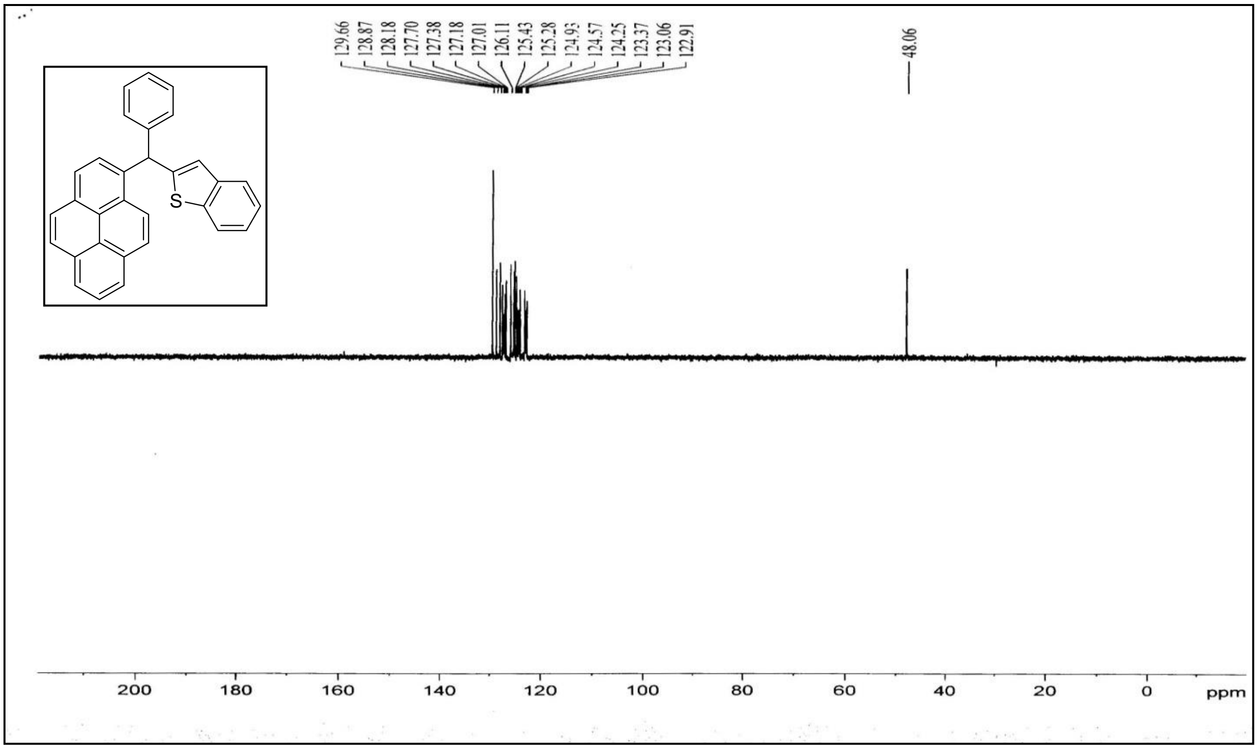

DEPT-135 spectrum of 2-(phenyl(pyren-1-yl)methyl)benzo[*b*]thiophene (**11r**).

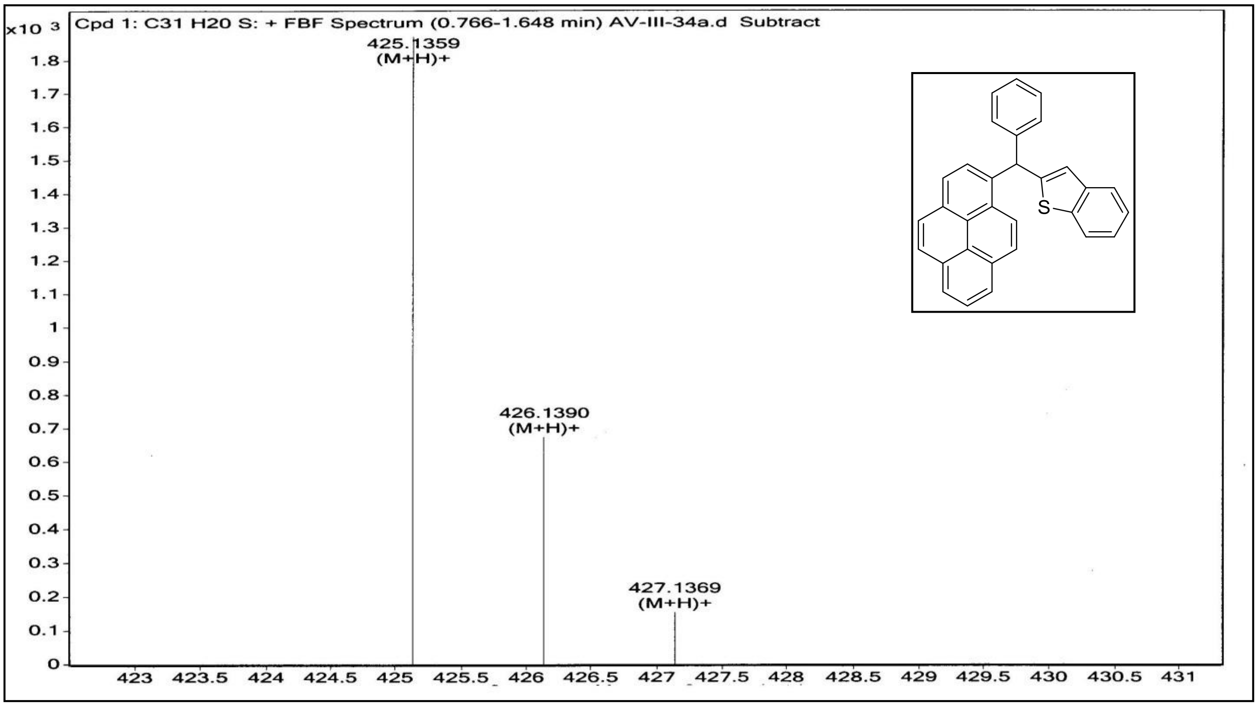

HRMS spectrum of 2-(phenyl(pyren-1-yl)methyl)benzo[*b*]thiophene (**11r**).

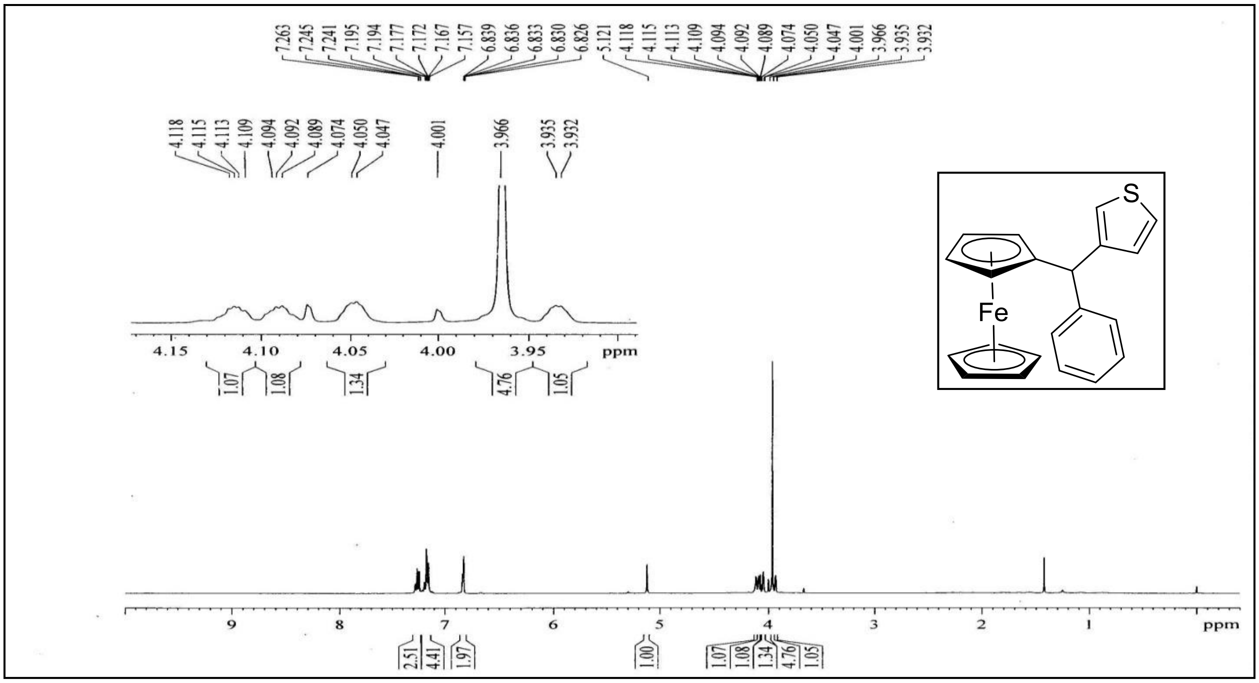

^{1}H NMR (400 MHz, $CDCl_3$ + CCl_4; 1:1) spectrum of 3-(phenyl(ferrocenyl)methyl)thiophene (**11s**).

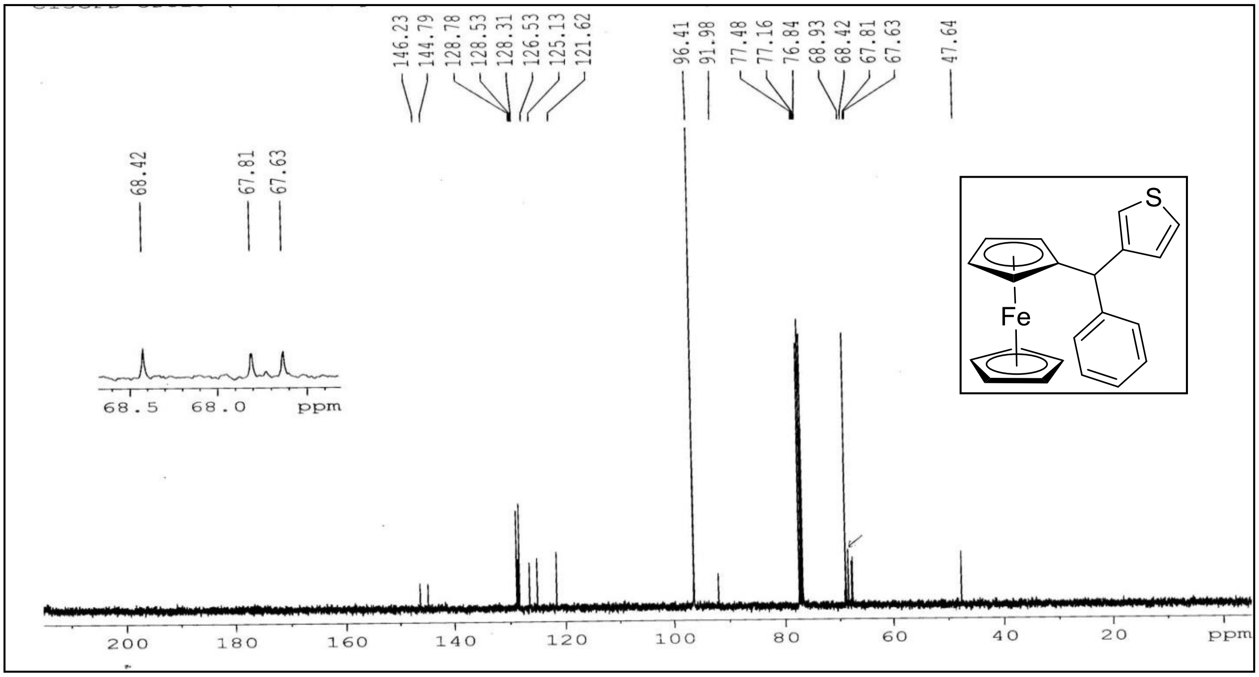

^{13}C NMR (100 MHz, $CDCl_3$ + CCl_4; 1:1) spectrum of 3-(phenyl(ferrocenyl)methyl)thiophene (**11s**).

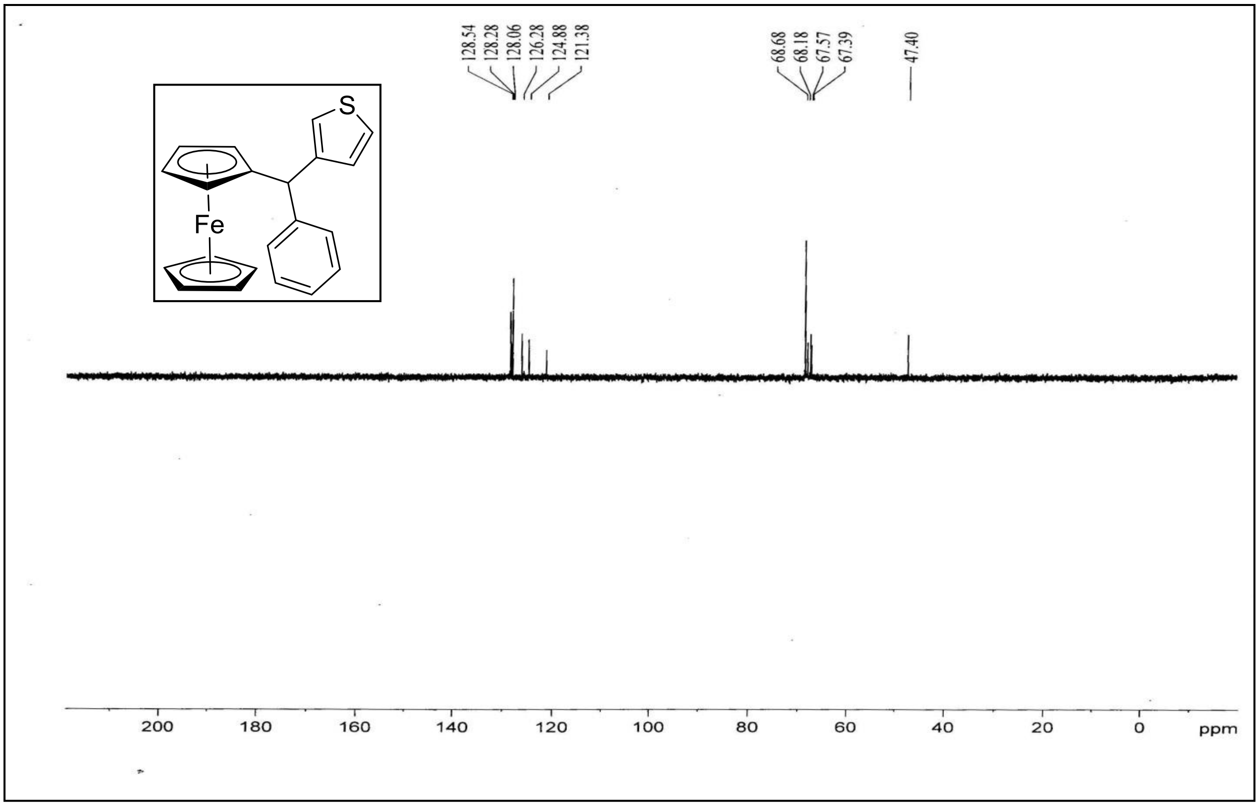

DEPT-135 spectrum of 3-(phenyl(ferrocenyl)methyl)thiophene (**11s**).

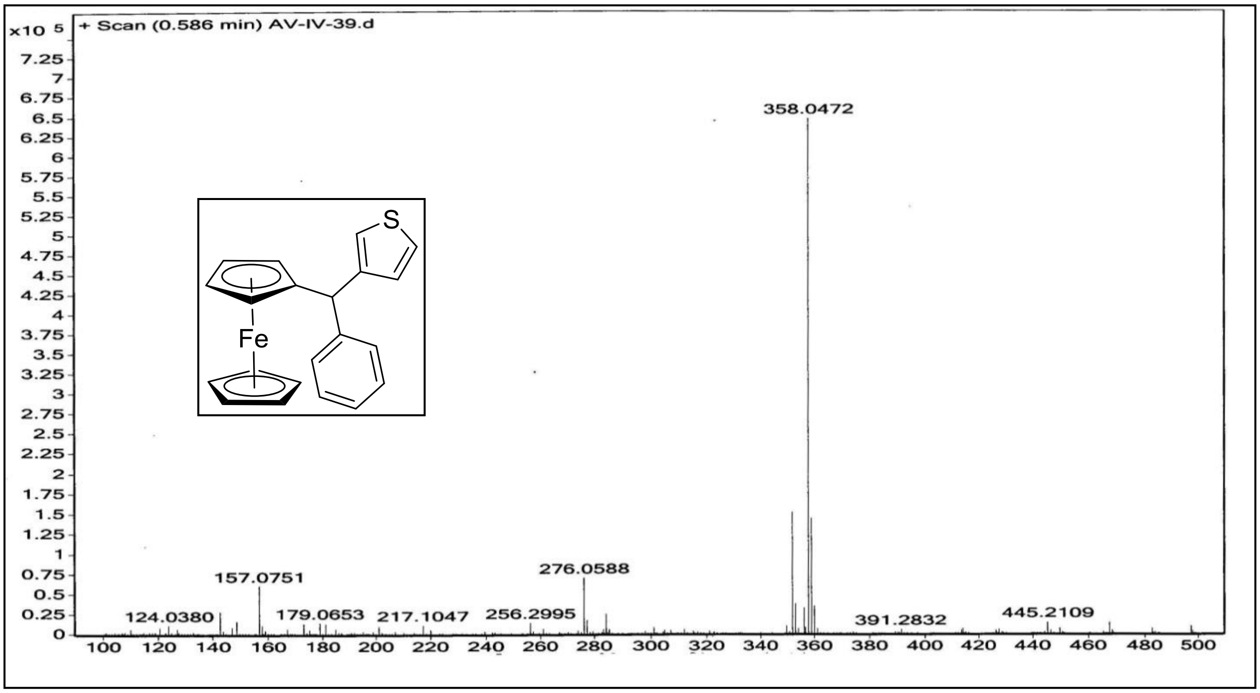

HRMS spectrum of 3-(phenyl(ferrocenyl)methyl)thiophene (**11s**).

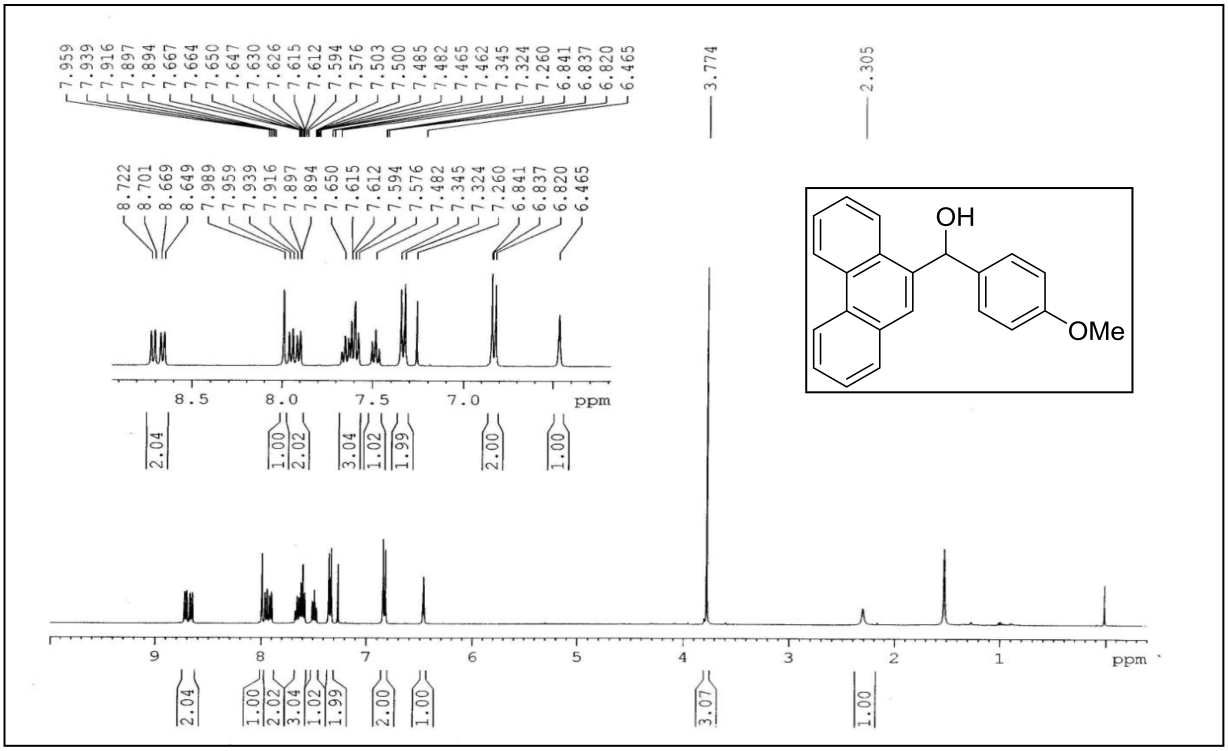

^{1}H NMR (400 MHz, $CDCl_3$ + CCl_4; 1:1) spectrum of (4-methoxyphenyl)(phenanthren-9-yl)methanol (**20**).

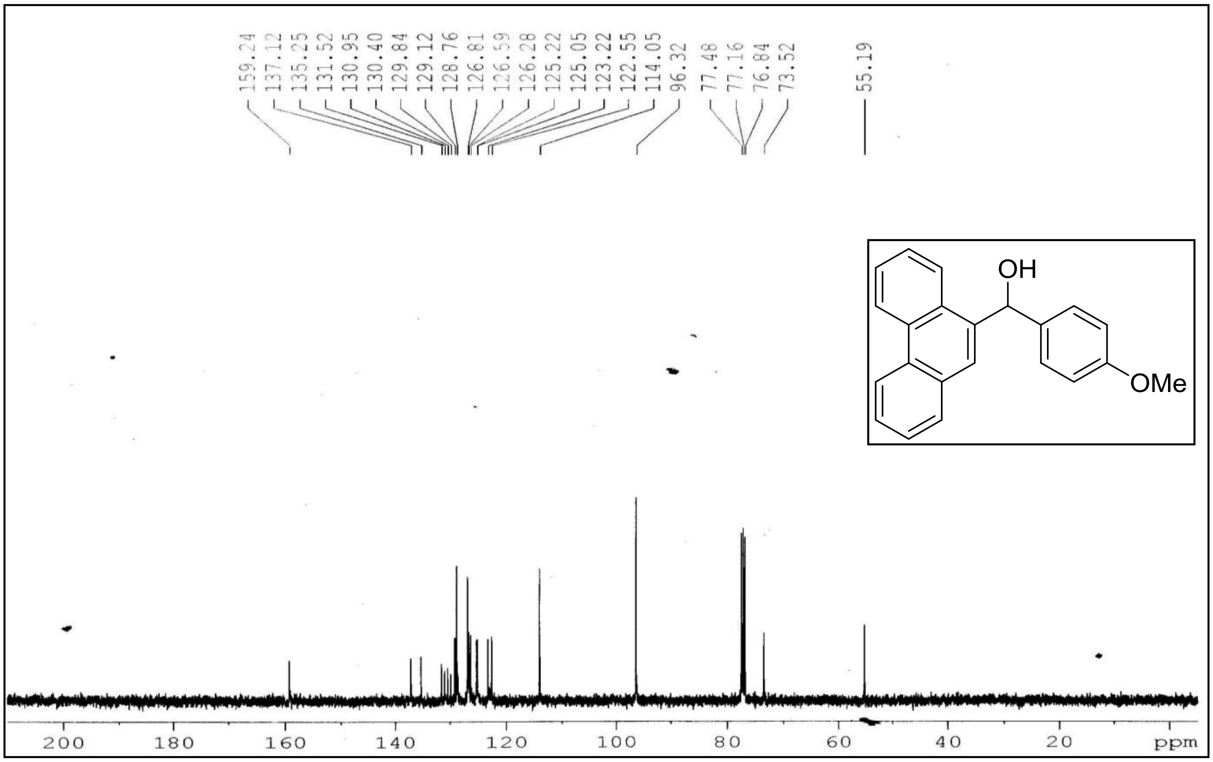

^{13}C NMR (100 MHz, $CDCl_3$ + CCl_4; 1:1) spectrum of (4-methoxyphenyl)(phenanthren-9-yl)methanol (**20**).

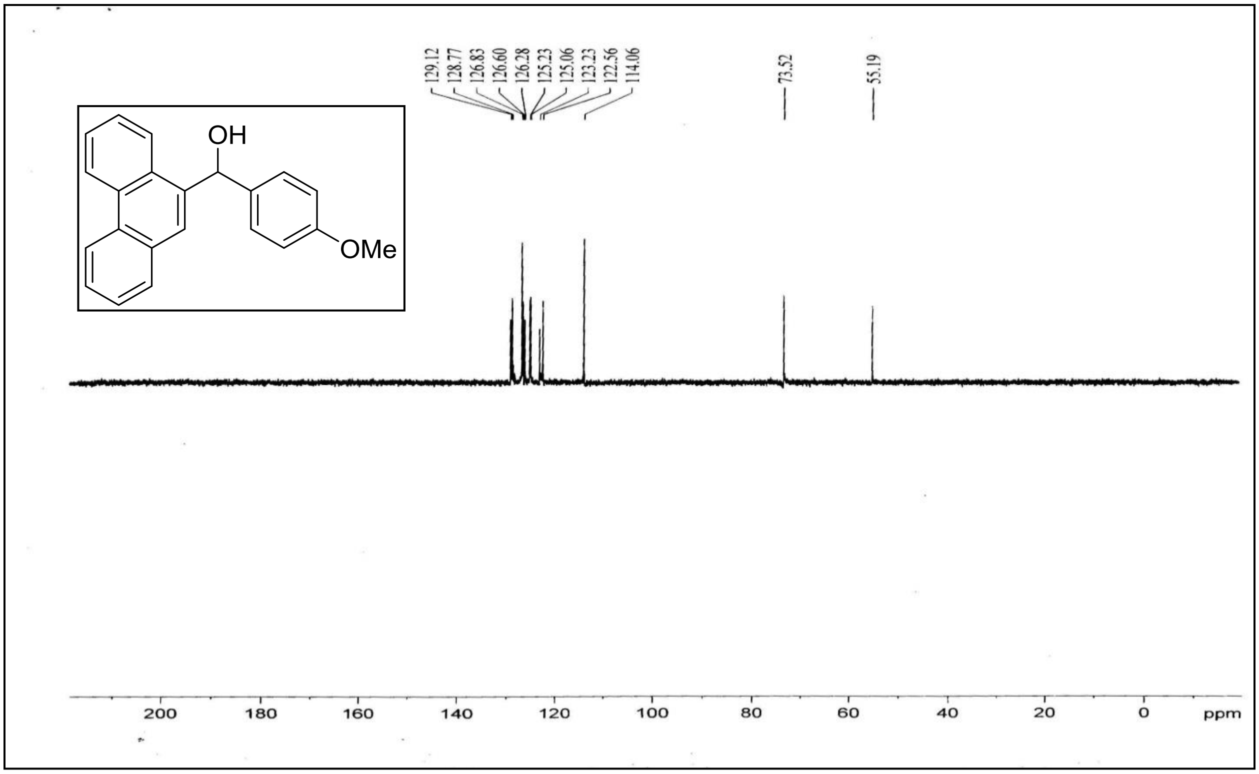

DEPT-135 spectrum of (4-methoxyphenyl)(phenanthren-9-yl)methanol (**20**).

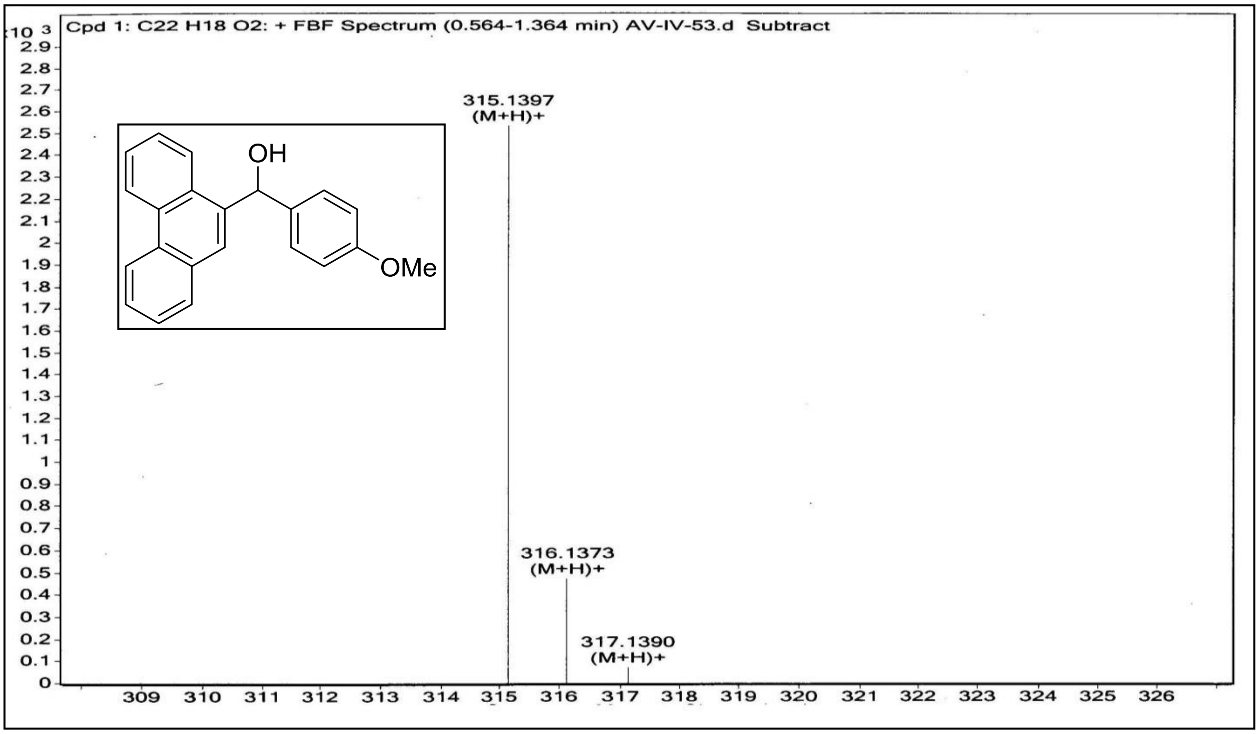

HRMS spectrum of (4-methoxyphenyl)(phenanthren-9-yl)methanol (**20**).

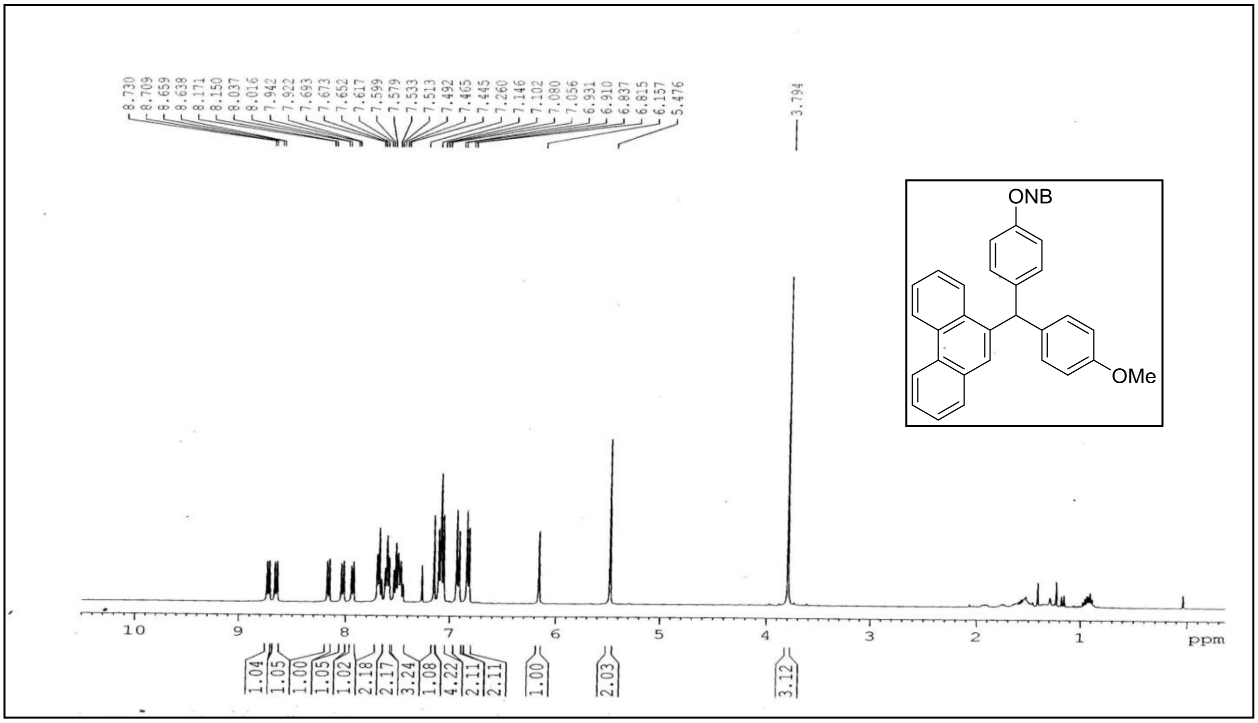

^{1}H NMR (400 MHz, $CDCl_3$ + CCl_4; 1:1) spectrum of 9-((4-methoxyphenyl)(4-((2-nitrobenzyl)oxy)phenyl)methyl)phenanthrene (**21**).

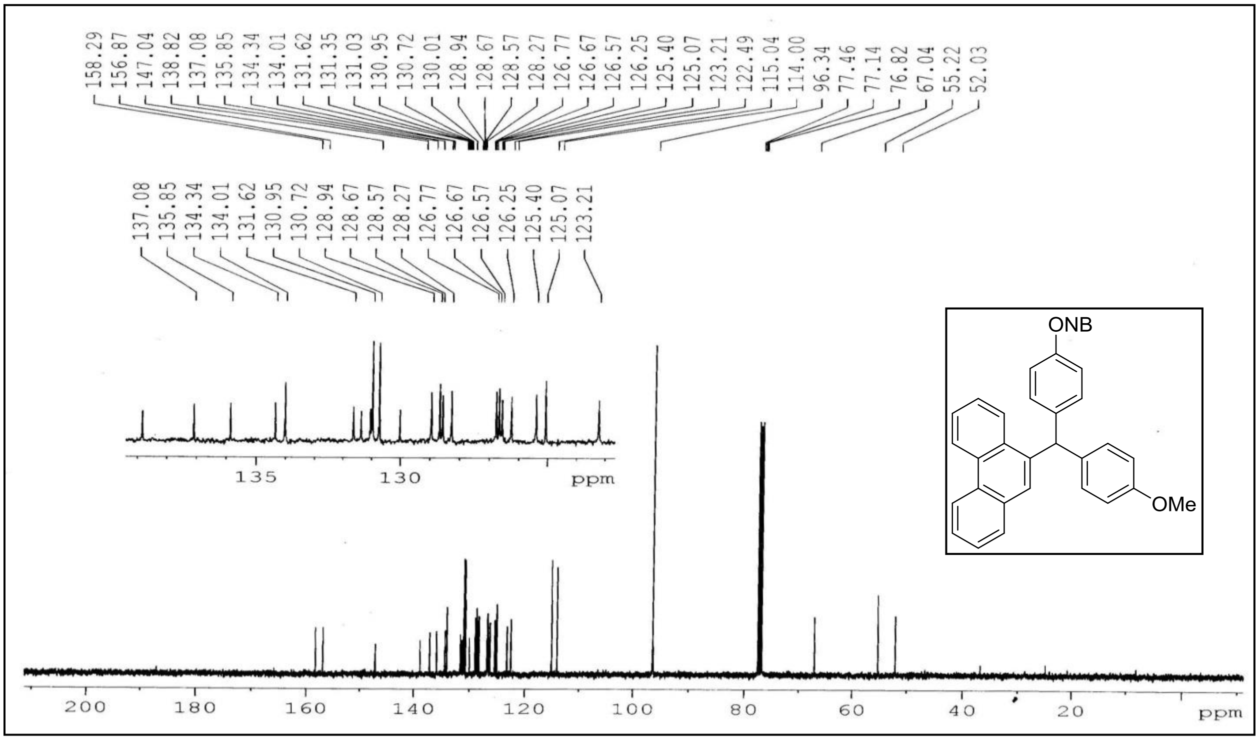

^{1}H NMR (400 MHz, $CDCl_3$ + CCl_4; 1:1) spectrum of 9-((4-methoxyphenyl)(4-((2-nitrobenzyl)oxy)phenyl)methyl)phenanthrene (**21**).

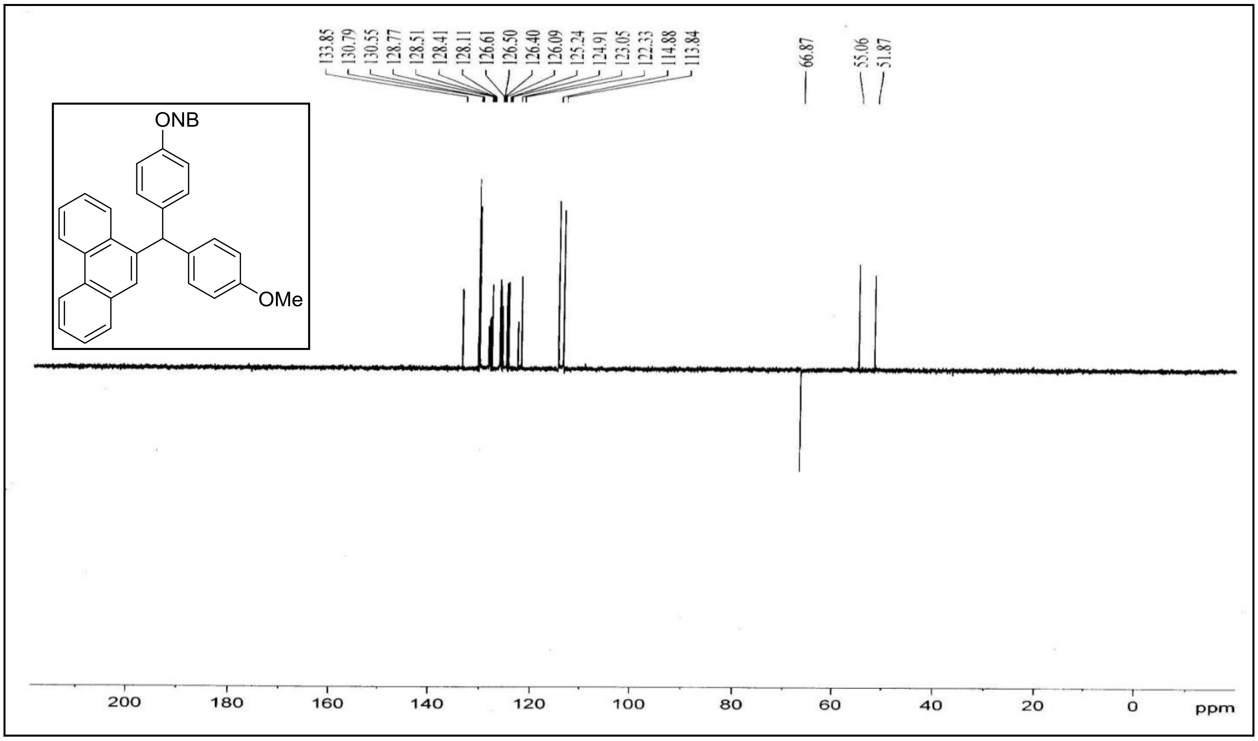

DEPT-135 spectrum of 9-((4-methoxyphenyl)(4-((2-nitrobenzyl)oxy)phenyl)methyl)phenanthrene (**21**).

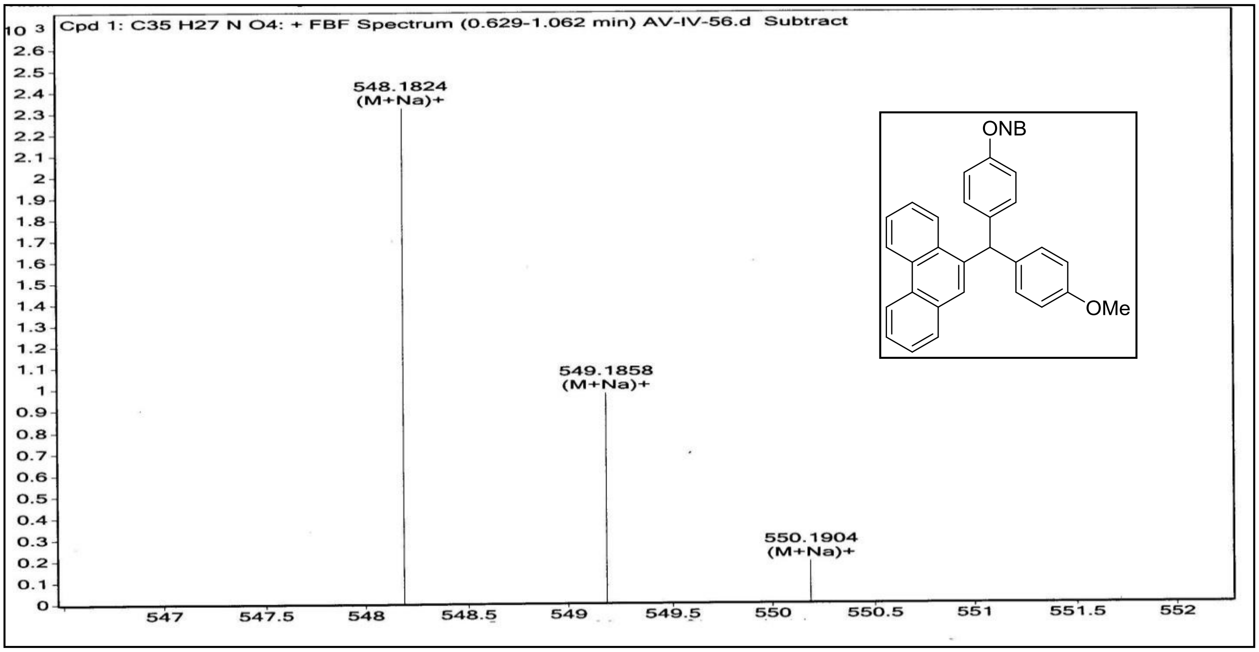

HRMS spectrum of 9-((4-methoxyphenyl)(4-((2-nitrobenzyl)oxy)phenyl)methyl)phenanthrene (**21**).

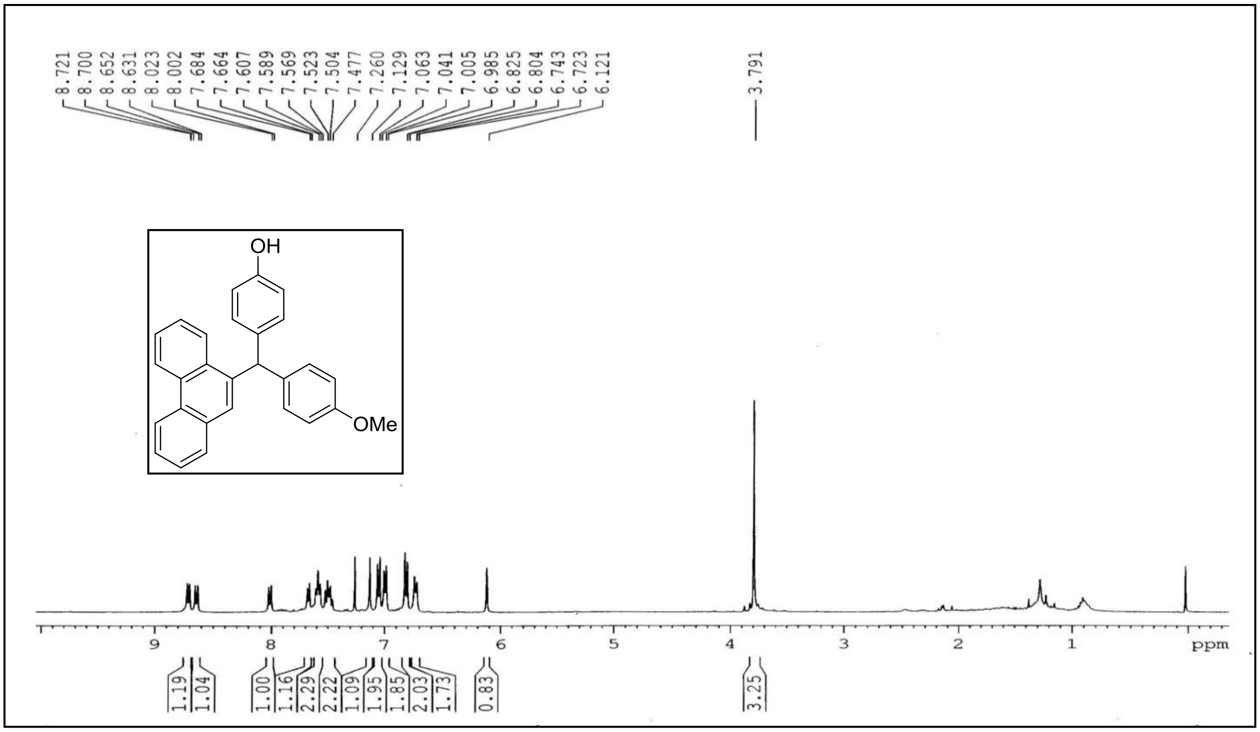

^{1}H NMR (400 MHz, $CDCl_3$ + CCl_4; 1:1) spectrum of 4-((4-methoxyphenyl)(phenanthren-9-yl)methyl)phenol (**22**).

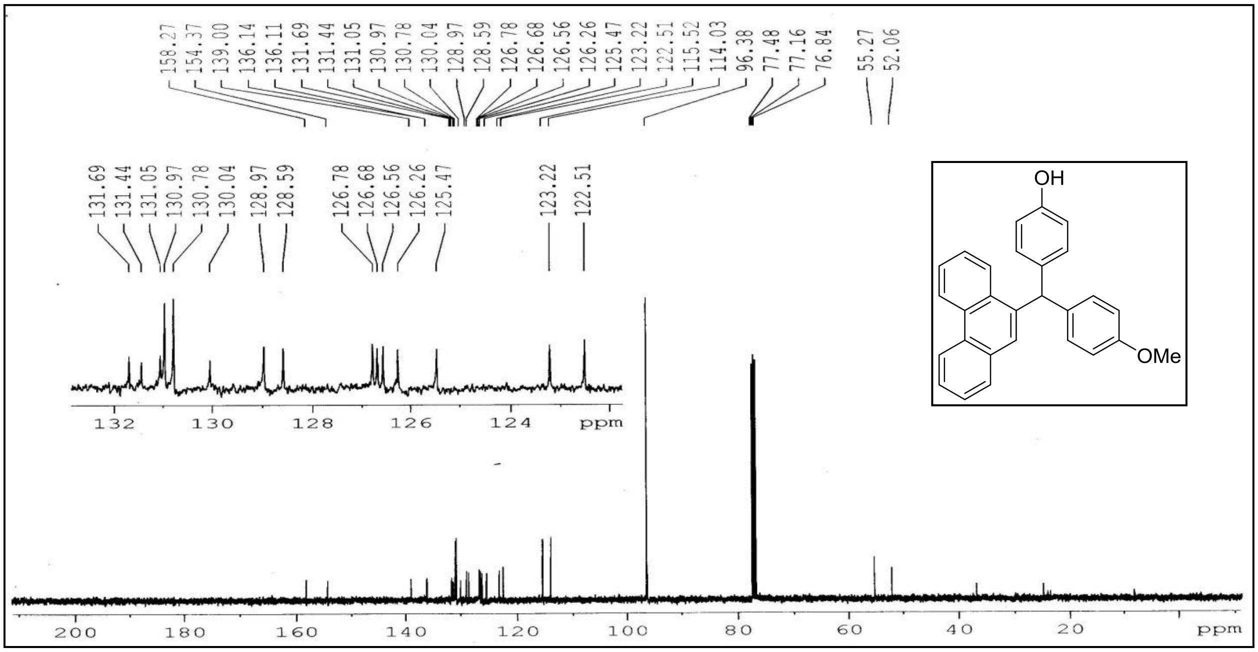

^{13}C NMR (100 MHz, $CDCl_3$ + CCl_4; 1:1) spectrum of 4-((4-methoxyphenyl)(phenanthren-9-yl)methyl)phenol (**22**).

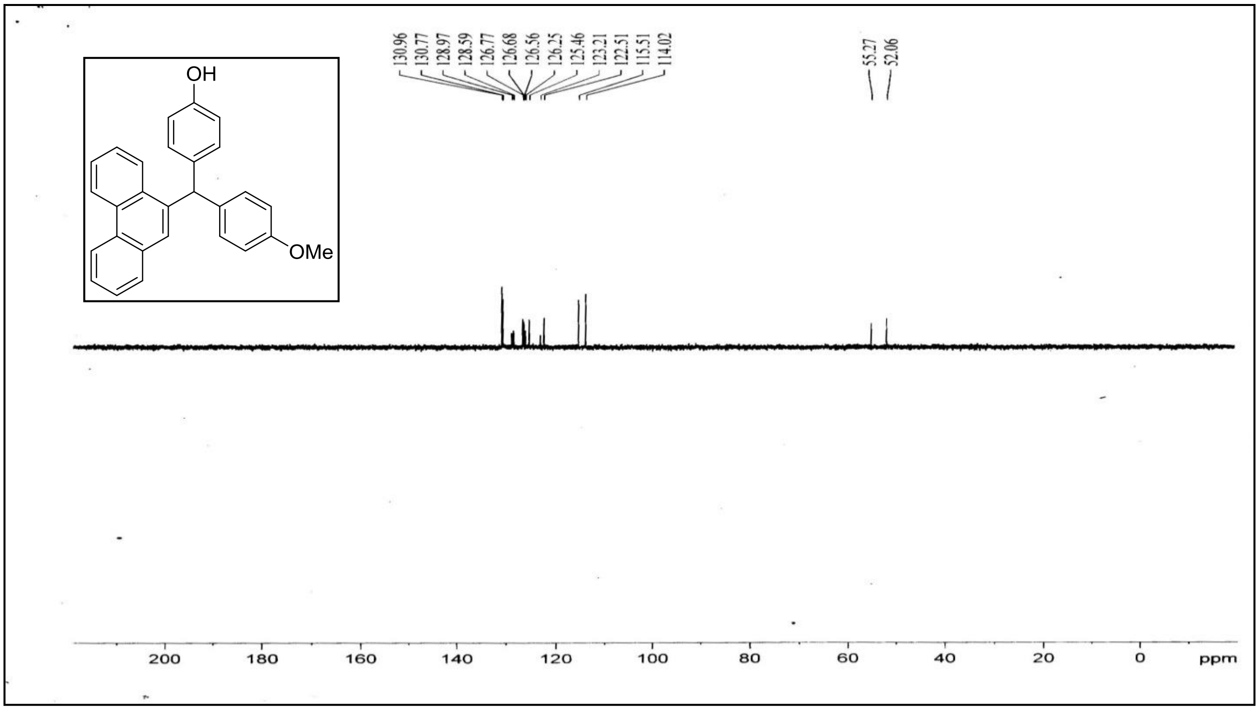

DEPT-135 spectrum of 4-((4-methoxyphenyl)(phenanthren-9-yl)methyl)phenol (**22**).

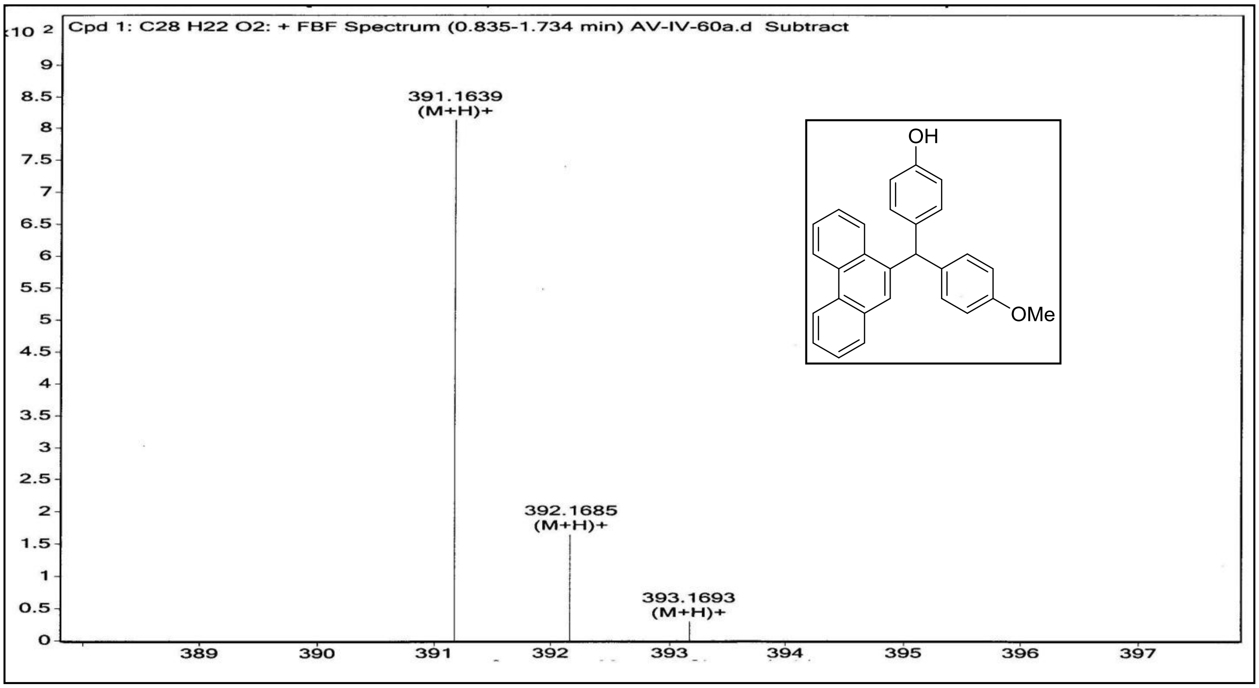

HRMS spectrum of 4-((4-methoxyphenyl)(phenanthren-9-yl)methyl)phenol (**22**).
